# A Review on Stimuli-Actuated 3D Micro/Nanostructures for Tissue Engineering and the Potential of Laser-Direct Writing via Two-Photon Polymerization for Structure Fabrication

**DOI:** 10.3390/ijms232214270

**Published:** 2022-11-17

**Authors:** Bogdan Stefanita Calin, Irina Alexandra Paun

**Affiliations:** 1 Center for Advanced Laser Technologies, National Institute for Laser, Plasma and Radiation Physics, 077125 Magurele, Romania; 2 Faculty of Applied Sciences, University Politehnica of Bucharest, 060042 Bucharest, Romania

**Keywords:** 3D micro/nanostructure, tissue engineering, laser-direct writing

## Abstract

In this review, we present the most recent and relevant research that has been done regarding the fabrication of 3D micro/nanostructures for tissue engineering applications. First, we make an overview of 3D micro/nanostructures that act as backbone constructs where the seeded cells can attach, proliferate and differentiate towards the formation of new tissue. Then, we describe the fabrication of 3D micro/nanostructures that are able to control the cellular processes leading to faster tissue regeneration, by actuation using topographical, mechanical, chemical, electric or magnetic stimuli. An in-depth analysis of the actuation of the 3D micro/nanostructures using each of the above-mentioned stimuli for controlling the behavior of the seeded cells is provided. For each type of stimulus, a particular recent application is presented and discussed, such as controlling the cell proliferation and avoiding the formation of a necrotic core (topographic stimulation), controlling the cell adhesion (nanostructuring), supporting the cell differentiation via nuclei deformation (mechanical stimulation), improving the osteogenesis (chemical and magnetic stimulation), controlled drug-delivery systems (electric stimulation) and fastening tissue formation (magnetic stimulation). The existing techniques used for the fabrication of such stimuli-actuated 3D micro/nanostructures, are briefly summarized. Special attention is dedicated to structures’ fabrication using laser-assisted technologies. The performances of stimuli-actuated 3D micro/nanostructures fabricated by laser-direct writing via two-photon polymerization are particularly emphasized.

## 1. Introduction

The design and fabrication of 3D structures at micro- and nanoscale represent a continuous challenge for biomedical applications such as tissue engineering [1,2,3], controlled drug delivery [4], cell biology [5,6,7,8] and biomimetics [9,10]. There are several fabrication approaches that initially provided reasonable results, but each of their advantages has been counterbalanced by at least one drawback. For example, extreme ultraviolet lithography (EUVL), focused ion beam or e-beam lithography (FIBL or EBL) enabled very small feature sizes with spatial resolutions reaching below 10 nm [11,12,13], but they can only provide 2D or 2.5D structures, whereas, for 3D structures, they are able to produce only very simple architectures, involving multistep fabrication processes [14,15]. In the recent past, direct laser-writing fabrication methods attracted great interest in the fabrication of 3D complex micro and nanostructures with high spatial accuracy and reproducibility [16]. Further progress has been achieved through the fabrication of stimuli-responsive 3D structures of length scales ranging from micro- to nanometers, having complex and reproducible geometries. Such structures have been fabricated by a wide variety of techniques and proved a high efficiency for biomedical applications [3,4,5,6,7,8,9,10,11,12,13,14,15,16,17,18,19]. Recently, the fabrication of stimuli-responsive 3D micro/nanostructures using laser-assisted protocols emerged as a powerful tool able to surmount the drawbacks of other technologies [18,19,20].

Topographical stimulation addresses issues with general tissue engineering aspects, such as cell volumetric cell migration and proliferation [21,22], with a particular emphasis on avoiding the formation of a necrotic core [21] by fabricating microstructures with controllable and reproducible geometries (size, shape, porosity, etc.). Structures with appropriate porosity (over 85%) allow for cell volumetric migration that results in improved viability and porosity. Multilayered structures have been shown to induce a 3.5-fold increase in Alkaline Phosphatase (ALP) production and 2.3 fold increase in osteocalcin secretion [23]. Cell behavior can be further influenced by dual-scale structures, i.e., structures that have geometric features at different size scales such as micrometer scale and nanometer scale. For example, recent results indicate controllable and reproducible antiadhesive properties of dual-scale structures for applications such as anti-biofouling [24]. While topographical stimulation shows promising results in a laboratory environment, there are still key issues such as scaling fabrication procedures for larger-scale applications and a lack of clinical trials for applications such as bone tissue regeneration.

Mechanical stimulation via low-intensity pulsed ultrasounds has been approved by the FDA for bone fracture treatments since 1994 [25,26]. Recent research has shown improved results in the case of osteoblast-like cells, such as a 200% increase in ALP production and a 100% increase in osteocalcin secretion, due to synergistic effects of topology and mechanical stimulation [27]. Further studies regarding these synergistic effects are required and may provide enhanced therapeutic solutions for bone tissue regeneration.

Chemical stimulation of microstructures provides enhanced properties for tissue engineering, such as improved biocompatibility. Current 3D-printing technologies offer design flexibility that is appropriate for tissue engineering, yet the usability of fabricated microstructures is limited by the available materials. A compromise is represented by structure functionalization, i.e., coating microstructures with other biocompatible materials that enhance cell proliferation. Studies regarding structure functionalization are limited, despite the scientific interest in the matter. A recent study shows the result of using Initiated Chemical Vapor Deposition to coat microstructures with polylactic acid and acrylonitrile butadiene styrene, combined with several hydrophilic polymers, but results indicate an uneven coating and structural damage due to thermal gradients [28]. Dip-coating with natural materials, such as collagen, show promising results but coating heterogeneity remains unresolved [29].

One important issue where electric stimulation shows a promising potential is controlled drug delivery. More precisely, electrically active scaffolds used for bone tissue engineering can be coated with conductive materials, such as polypyrolle, and used to locally deliver anti-inflammatory drugs, such as dexamethasone. Moreover, electrically stimulated seeded structures show increased cellular activity, with results showing a 2.2-fold increase in ALP production [30]. Extensive in vitro research is still necessary to develop electrically active scaffolds for tissue engineering that can also deliver drugs locally, before in vivo research can be approached.

Magnetic stimulation is used as an approach to hasten bone tissue regeneration. Static magnetic fields have been found to accelerate tissue regeneration in osteoblast-like cells [31,32,33,34,35,36,37,38,39,40,41,42]. However, fabricating reproducible 3D microstructures that are magnetically responsive proved to be a challenge. One approach is coating microstructures with magnetic nanoparticles [37]. Recent studies show promising results with 3D-printed microstructures using composite materials made of photosensitive resins with magnetic nanoparticles homogeneously dispersed throughout [31]. Despite the advantages of magnetic stimulation, there is limited research conducted on magnetically active 3D microstructures for tissue engineering, mostly due to the difficulty in fabricating reproducible architectures that contain an appropriate amount of magnetic nanoparticles, with a homogeneous volumetric distribution.

This review addresses the state of the art concerning 3D micro/nanostructures for tissue engineering applications, with particular emphasis on structures’ actuation for improving their functionality. The paper also highlights the potential of laser-direct writing techniques for the fabrication of 3D micro/nanostructures. Aside from the challenging topic of tissue engineering, the field of stimuli-actuated 3D structures has a broad range of applications, from medical use [43] to chemistry [44], and it is going towards multidisciplinary areas such as micro- and nanofluidics [45,46].

In this review, we will present an overview of existing methods for the fabrication of 3D micro/nanostructures that can be stimulated by different types of stimuli (topographic, mechanic, electrical or magnetic stimuli). Examples of such structures and their applications will be presented. Materials that can be processed with laser-direct writing technologies represent an exceptionally broad domain, yet the technologies presented will be applicable to photopolymeric resins, be it cross-linking (e.g., SU-8), chain polymerization (e.g., IP photoresists, Ormocore), composite materials (e.g., various ratios of photopolymer and additional materials such as nanoparticles), photosensitive glass (e.g., Foturan), organic materials (e.g., hydrogels, biomolecules, etc.) and others [17,47,48,49,50,51,52,53,54,55,56]. It is worth mentioning that these technologies can also be employed in a multistep fabrication procedure such as providing masks for UV lithography. We will emphasize how the synergistic action between the fabrication methods and the stimuli-responsive materials employed for structure fabrication open up new perspectives for 3D structures stimulation at micro/nanoscales for tissue engineering.

A broad analysis of different techniques for fabricating 3D micro/nanostructures, as well as the means for improving their functionality through stimulation with the above-mentioned stimuli, is provided. Moreover, the study addresses the main types of stimuli that have been applied to 3D micro/nanostructures with the purpose to improve their functionality for biomedical applications. 

Particular attention is dedicated to laser-assisted technologies that emerge as powerful tools for precise, reproducible and versatile fabrication of 3D micro/nanostructures. The review also identifies different approaches for building intrinsically active and passive, i.e., postprocessing-activated, 3D microstructures. The potential of Laser-Direct Writing via Two-Photon Polymerization (LDW via TPP) in this manufacturing field will be particularly emphasized. 

## 2. Laser Assisted Fabrication Methods

Tissue engineering is focused on the development of biological replacements for living tissues and organs that reduce the need for compatible donors and improve post-implantation results [43]. This is generally achieved through 3D micro/nanostructures that are biocompatible, sometimes biodegradable, and that provide appropriate 3D environments for the cells to attach and interconnect in 3D architectures similar to the native tissues [5,14,15,20]. After cell seeding, the scaffolds are implanted at the desired site inside the body, where the cells continue to grow and finally replace the damaged tissue; in the end, the whole construct (scaffold and cells) is integrated in the native tissue.

Recent advances in the fabrication of such 3D micro/nanostructures using 3D printing technologies have determined an increasing number of researchers to use laser technologies for biomedical applications, such as regenerative medicine and tissue engineering [5,6,7,8,9,10,11,12,13,14,15]. 

In particular, due to laser-assisted 3D printing technologies of biomaterials, numerous biomedical applications are currently being developed, from tissue engineering to the development of new drugs and diagnostics. Laser-assisted techniques, such as Laser Induced Forward Transfer (LIFT) and stereolithography (SLA), have been successfully used for fabricating two-dimensional microstructures with various geometries that could be used to influence cell bio-dynamics (e.g., cells attachment, cells proliferation, etc.) [57,58]. However, these structures have shown several limitations, the most important being reduced cell viability and the lack of 3D inter-cellular connections similar to living tissues, which determined the formation of unwanted necrotic cores [3]. 

**Figure 1:**
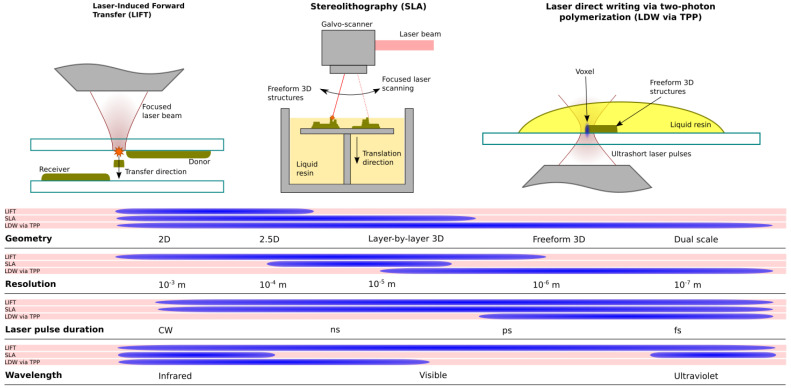
Schematic representation of three of the most common laser-direct writing technologies used for biomedical applications and tissue engineering, and their general characteristics and requirements.

Laser-assisted 3D printing technologies come as a spectrum, as particular experimental setup components and devices can be mixed and matched in order to achieve the desired experimental conditions and fabrication characteristics, and satisfy material requirements. However, we present three of the most commonly encountered concepts for laser-direct writing technologies employed in microstructure fabrication for biomedical applications, with an emphasis on tissue engineering (see Figure 1). More precisely, as mentioned above, these are LIFT, SLA and LDW via TPP. 

If we consider geometric characteristics, in general, LIFT is used for 2D and 2.5D printing, SLA is used for 3D printing but is limited by a layer-by-layer approach, and LDW via TPP can achieve freeform 3D printing as well as dual-scale structures. Dual scale refers to those structures that have elements of different scales, i.e., micrometer scale and nanometer scale, with a controllable geometry. 

The printing resolution is determined by the effective laser spot size and the targeted material. LIFT allows for an extended choice of resolution that can accommodate various requirements, i.e., for printing larger areas, a larger spot size is preferred. However, in the context of tissue engineering, LIFT is generally used to print organic materials that limit the usable laser spot size, i.e., a smaller laser spot size may be used but the material will not transfer appropriately. SLA is usually realized using a galvo-scanner, which determines the limits of the usable laser spot size. Large working distances provided by the galvo-scanner mean faster processing of larger areas, but limit the minimum laser spot size to a diameter of several micrometers. LDW via TPP, however, is limited by the polymerization process and material properties. Since the main laser-matter interaction mechanism is two-photon absorption, resolutions below the diffraction limit are achievable.

Both LIFT and SLA rely on linear absorption, which technically means any incident laser pulse duration can be used (and has been reported throughout the years), as long as other parameters, such as laser fluence, are appropriate. LDW via TPP requires ultrashort pulses. Nonlinear optical effects, such as two-photon absorption, may appear under various conditions and do not necessarily require ultrashort pulses. However, for LDW via TPP specifically, one of the core characteristics is processing below the diffraction limit through means of a volume pixel (voxel) formation, which is noticeable for laser pulses with a duration below ~10 ps. The pulsed laser repetition rate is not approached here as it determines processing speed but does not technically restrict structure fabrication itself.

Incident laser wavelength intervals are determined by material characteristics. For LIFT, the incident laser wavelength must be chosen so that it can detach the material from the donor substrate with minimal or no damage to the deposited material itself. Wavelengths used for SLA are determined by the polymerization mechanism, i.e., infrared wavelengths are used for thermally activated resins, and UV for photo-sensitive resins. LDW via TPP is restricted to infrared and part of the visible spectrum, as any thermally activated resins would not allow for the achievable resolution using this technology.

The technique known as Laser Induced Forward Transfer (LIFT) involves the transfer of materials from a donor substrate to a receiver substrate by means of laser irradiation [59]. This technique is a solvent-free non-contact 2D printing technology with many applications in both industry and academia [59]. The laser beam breaks off the material from the donor substrate and projects it forward towards the receiver substrate. As mentioned previously, the deposited material volume (voxel, as mentioned above). One of the main advantages of this method is the fact that it can be used with a diverse selection of materials, both solids and liquids [59]. Biomolecules, for example, are usually handled in aqueous solutions and can be efficiently deposited using LIFT. During laser processing, only a small amount of material is evaporated from the donor substrate, which in turn, means that biomolecules can be laser-printed using LIFT in spite of their generally fragile nature [59]. As a consequence of LIFT’s generally non-destructive characteristics with regard to the donor material, this method can be used for bioprinting. In the case of bioprinting, cells are usually handled in high-viscosity hydrogel solutions, that allow for various cell types and biological factors to combine so that the resulting structure mimics the heterogeneity and the composition of living tissues [60]. Over the years, various cell types have been successfully deposited using LIFT, among which we can mention B35 neuronal cells, bovine aortic endothelial cells, fibroblasts, osteoblast-like cells, Escherichia coli, endothelial cells, stem cells and others [59]. LIFT can be used to fabricate 3D structures directly using cells or cell combinations. However, in spite of these capabilities, there are some important drawbacks. Using cells for the whole fabrication protocol makes the activity sensitive to experimental conditions which can affect the cell viability. Moreover, the process allows only layer-by-layer fabrication, which increases the cost and fabrication time. Another issue is that the incident laser radiation must be used for a particular cell phenotype. For example, Barron et al. [61] managed to fabricate 3D osteosarcoma structures, but they used an incident laser radiation of 193 nm. While cell viability tests provided good results, there were some concerns about long-term radiation damage due to intense UV exposure. 

Another popular laser-direct writing technique used for 3D printing is stereolithography (SLA) [62]. This method uses a laser beam directed through a galvo-scanner and appropriate optics to irradiate a photopolymer and to construct a 3D microstructure in a layer-by-layer manner. 

Stereolithography allows for the fabrication of 3D structures over a large-scale interval, from micrometer scale to macroscopic structures [62]. This range allows for a fabrication process that can be adapted to specific patient requirements for applications such as complex surgical interventions, matrix-assisted implant fabrication and custom-designed products such as hearing aids [63]. More recently, stereolithography has been improved as a fabrication technology through the development of biodegradable photo-processable materials for implants. Furthermore, stereolithography can be used with advanced imaging techniques such as magnetic resonance imaging (MRI) and computed tomography scan [64]. Laser-direct writing of implantable devices, such as biodegradable scaffolds used in tissue engineering, is also improved by the development of new photopolymeric materials. A significant step forward for stereolithography was determined by the introduction of modified natural polymers, such as hydroxyapatite-based composites and cell-containing hydrogels [65]. Resolution-wise, stereolithography has been usually used to fabricate microstructures for tissue engineering with pore sizes ranging in the hundreds of micrometer range [66], although structures with smaller spatial features can be obtained. For example, Brandi et al. [67] report a minimum polymerization depth of 300 nm, although absorption depth is the principal determining factor of the aspect ratio of obtainable structures, and lateral spatial features are still determined by the laser spot size and wavelength. The capability of fabricating custom 3D microstructures with reproducible geometries, using various biocompatible and biodegradable materials such as polycaprolactone, poly(propylene fumarate), trimethylene carbonate and others [66], provides a great potential for stereolithography for biomedical applications, in particular for tissue engineering. However, the fact that this method is based on linear absorption, i.e., photopolymerization takes place at the surface of the liquid photoresist, limits the capabilities and resolution. In particular, laser spot size dictates the achievable resolution, and therefore the spatial features of the obtained 3D microstructures, and the fact that laser-matter interaction occurs at the surface of the photoresist limits the geometries that can be fabricated.

Linear or single-photon absorption is the principal laser-matter interaction mechanism that locally generates the polymerization process in the photopolymeric resin. While stereolithography is a fast, cheap and reliable method for 3D microfabrication, it has some limitations, the most important being the diffraction-limited spatial resolution and the necessity of liquid photopolymeric resins (for 3D structures) [66].

A very popular photopolymerization technique based on laser-direct writing involves two-photon absorption (usually referred to as Laser-direct Writing via Two-Photon Polymerization—LDW via TPP) [68]. This laser-writing technique retains the most important advantages of stereolithography, i.e., arbitrary 3D structures, but overcomes its limitations, i.e., resolution below the diffraction limit and more freedom in terms of design as the laser-matter interaction that is not limited to the surface of the photoresist. The photopolymeric resin is irradiated with infrared ultrashort laser pulses (usually of the order of 10^2^ fs, but using ps pulses have been reported) that are focused using a microscope objective with an appropriate magnification. The photoresist is transparent to the laser wavelength, while also showing strong absorption to the second harmonic. As such, the photochemical reactions take place close to the focal point of the laser beam, where the laser intensity exceeds a certain threshold, after which two-photon absorption processes become the main laser-matter interaction mechanism. Usually, photoinitiator molecules are targeted in a liquid photopolymer, that is ionized when irradiated with the second harmonic and generates free radicals. These free radicals generate and maintain the chain polymerization processes [69]. Using Gaussian beams, a spatial resolution below the diffraction limit can be obtained, while also containing the polymerization process within a specific volume into the photoresin. This method allows for significantly better spatial resolution than MAPLE, LIFT or stereolithography, useful for designing and fabricating complex nano- and microstructures, in both solid and liquid photoresists, albeit with lower processing speeds and total volumes when compared to stereolithography.

Another limitation is that the applicability of LDW via TPP is restrained by the range of materials available for structures’ fabrication [68,69], which are generally limited to photopolymerizable polymers/resins. Although these materials have been strongly improved for increasing the spatial resolution of the laser-imprinted structures, this is by far not enough to make the obtained structures useful for biomedical applications. At present, there is a pressing need to make those passive structures active. This can be done by actuating the 3D structures with different types of stimuli (either via intrinsic stimuli such as topography, or by externally applied stimuli such as mechanical, chemical, electric or magnetic). In this context, LDW via TPP technology is extending its original role of producing backbone, i.e., passive structures than can be functionalized only through post-fabrication procedures, towards the fabrication of intrinsically active structures that are capable to transduce diverse stimuli such as topographic, mechanic, chemical, electric or magnetic [70,71,72,73].

To date, the fabrication of stimuli-responsive 3D micro/nanostructures for biomedical use relies on direct and indirect methodologies. In the indirect methodology, 3D micro/nanostructures are first fabricated as “skeletons” and become active through postprocessing procedures, e.g., by dip-coating or by external application of mechanic, electric or magnetic stimuli [74,75,76,77,78]. On the other side, in direct methodologies, the 3D micro/nanostructures are active right after their fabrication process, because active materials are incorporated directly into the backbone 3D micro/nanostructures, during the fabrication process [79,80,81,82]. Direct methodologies generally provide a more time and cost-efficient approach to microstructure fabrication. Moreover, direct methodologies are often associated with a higher degree of reproducibility, due to fewer fabrication steps involved. However, direct methodologies are highly dependent on material properties and development. Therefore, indirect technologies are more often used as they allow for a significantly broader material choice and higher fabrication flexibility, albeit with the associated disadvantages: slower fabrication, higher cost and complex multistep fabrication techniques, which lower reproducibility.

**Figure 2:**
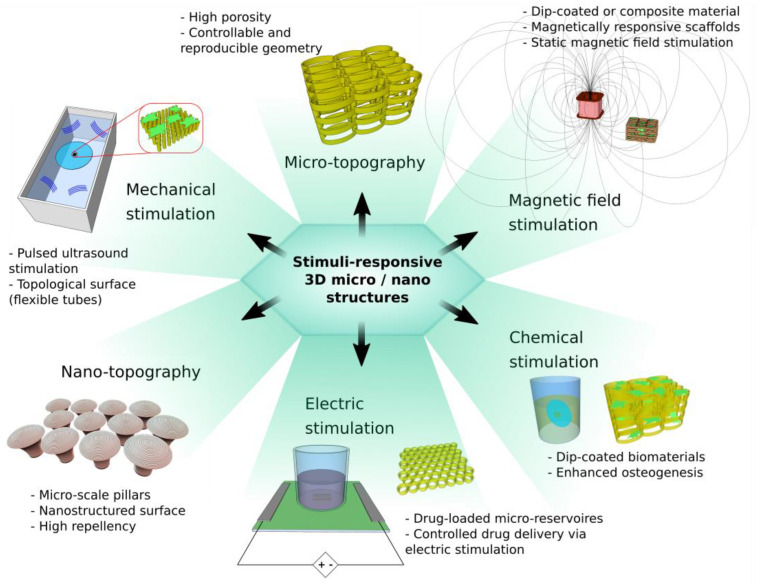
Schematic representation regarding the actuation of 3D micro/nanostructures using different types of stimuli (topographical, chemical, mechanical, electric and magnetic) for tissue engineering applications.

In the following section, a detailed description of LDW via the TPP method is provided. This will be followed by several subsections that will address a particular category of stimuli-responsive 3D micro/nanostructures for biomedical use. Figure 2 illustrates a schematic representation regarding the actuation of 3D micro/nanostructures using different types of stimuli (topographical, chemical, mechanic, electric and magnetic). The most relevant studies on the topic will be summarized and sustained by a comprehensive list of references.

## 3. Laser-direct Writing via Two Photon Polymerization

Often also known as 3D Lithography, Laser-Direct Writing via Two-Photon Polymerization (LDW via TPP) is a direct writing technique where two-photon absorption is the principal laser-matter interaction mechanism [83]. This method is used for fabricating high-resolution, complex micro/nanostructures from biocompatible and/or biodegradable polymers. The photopolymer is irradiated with ultrashort laser pulses centered on a wavelength for which the resin is transparent, but shows high absorption for the second harmonic. Pulse duration and energy are important in order for the two-photon absorption threshold to be exceeded only in a defined prolate volume, i.e., the voxel, whose physical significance was described in the above paragraphs, situated in the focal point of the focusing optics. The trajectory of the voxel is used to describe the geometry of the desired micro/nanostructure to be imprinted. LDW via TPP has shown significant advantages when compared to similar methods such as LIFT or stereolithography, among which the most important are: resolutions below the diffraction limit can be achieved and the micro/nanostructures can be fabricated via freely moving the voxel in 3D, i.e., it is not bound to a layer-by-layer fabrication method and, as a consequence, it allows the fabrication of practically any desired 3D architecture, with no geometrical constraints [68,69,70,71,72,73].

While LDW via TPP can be used with both solid and liquid photoresists based either on cross-linking or chain polymerization processes, LDW via TPP uses either liquid polymeric photoresists based on chain polymerization, or solid photoresists based on cross-linking. The use of solid photoresists requires pre- and post-exposure processing steps such as spin coating and multistep pre-baking and post-baking, which lower the resolution, limit the height of the micro/nanostructures and increase the fabrication time. On the other side, the used of liquid chain polymerization photoresists, is significantly simpler. The general fabrication procedure for LDW via TPP using liquid photoresists (i.e., where the non-irradiated material is removed) starts with the drop-casting of a drop of photoresist on an appropriate (generally glass) substrate, followed by the irradiation of the photoresists with the focused laser beam and finally the development through the immersion of the sample in an appropriate solvent that washes away the non-polymerized resin. The use of a liquid photoresist requires that micro/nanostructure fabrication begins at an attachment point, i.e., on the substrate or other existing fixed structures.

Going into more detail [68,69,70,71,72], the liquid chain-reaction photoresists are comprised of several molecules, among which the most important are the photoinitiator (PI), the monomer molecules (M), and other substances that control properties such as chemical stability, viscosity, density, absorption, etc. The incident laser pulses are most often centered on a wavelength in the near-IR range and have a Gaussian intensity distribution. As such, commonly encountered laser sources are Er:doped fiber or, less common, Ti:sapphire laser systems [84], delivering pulses with a wavelength centered on 780 nm (second harmonic for ER:doped laser medium) or 800 nm (Ti:sapphire laser medium). While the photoresist must be transparent for the incident laser wavelength, the PI must strongly interact with the second harmonic. If appropriate conditions are met, i.e., the laser intensity exceeds the two-photon absorption threshold, the PI molecules are ionized and generate free radicals (known as the initiation step). The free radicals then form a bond with a monomer molecule, resulting in a new molecule having active terminations (known as the propagation step). This molecule continues the process of bonding with other monomer molecules, until it bonds with another molecule that has active terminations (known as the termination step). The process results in randomly generated polymeric chains that intertwine and generate a solid polymeric material filling the voxel. The rest of the photoresist that was not irradiated is removed by immersing the sample in the appropriate solvent.

Solid photoresists, i.e., based on cross-linking, are usually used for 2D and simple 2.5D structures, more often involved in microfluidic applications, in the context of biomedical research. They offer great structural and mechanical resistance and can be processed over larger areas via lithographic methods. However, they are considerably limited in height when compared to liquid photoresists and require a more complex fabrication protocol, usually involving spin-coating, pre- and post-baking steps or even multistep development. This adds to costs, and fabrication time and reproducibility are lowered as fabrication complexity rises. Liquid photoresists, on the other hand, can be used for freeform 3D microstructures with high reproducibility, and allow for time and cost-efficient fabrication. Development of structures fabricated using liquid photoresists usually involves either immersion or washing with an appropriate solvent.

Usually, the laser-writing techniques are analyzed in terms of laser power and writing velocity. However, in the case of LDW via TPP, it is useful to discuss in terms of polymerization degree and energy dose [68,69,70]. That is because the resulting polymer is a conglomerate of intertwined polymeric chains with various lengths. The “polymerization degree” refers to the percentage of the monomer molecules, in the irradiated volume, that are part of a polymeric chain, as opposed to the monomer molecules that remained unattached. This percentage directly influences the quality and stability of resulting structures. A structure with a lower polymerization degree has a higher percentage of unpolymerized material in the irradiated volume. This unpolymerized material will be removed in the development step and the sample drying, which generates a phenomenon known as “polymer shrinkage”, in which the resulting polymer dries up and lowers its volume. Lowering the volume produces deformations and induces mechanical tension throughout the imprinted structure, which can result in the structure peeling off the substrate, breaking, bending, etc. Another possible defect directly influenced by the polymerization degree is the structure bending and welding, which is specific to nano-scale structures. The other factor considered important for LDW via TPP, namely the “energy dose”, is what determines the polymerization degree, which is a factor depending on both writing speed and laser power. The polymerization process requires a certain amount of time to finish, but it is faster if the photon density is higher, i.e., higher laser intensities. In other words, one can obtain similar structures with either higher laser power or writing speeds, or lower laser powers with lower writing speeds. The difference is the probability of the formation of defects within the 3D structure, which is a stochastic process. Photoresists can have small imperfections throughout their volume, either clumps of monomer molecules, (small polymeric chains as a result of exposure to natural light), or impurities that infiltrated during sample manipulation. These imperfections, be it transparent or not, can generate near-field intensification of incident laser radiation. The laser intensity should exceed the two-photon absorption threshold for the polymerization to take place, but if the intensity is too great, it can break molecules further and generate gaseous components, resulting in microbubbles (usually called microexplosions). These microbubbles affect both the neighboring polymer structure, as well as the laser focusing. Focusing and near-field intensification effects can also be induced by existing polymeric structures, if the right conditions appear (i.e., the resulting polymer is transparent but has a higher refractive index and thus focuses more on the incident laser radiation). The probability of microexplosions increases proportional to the incident laser power. In contrast, using lower laser powers and writing speeds, increases the probability of obtaining a defect-free microstructure, but the fabrication time increases significantly, depending on the geometry.

**Figure 3:**
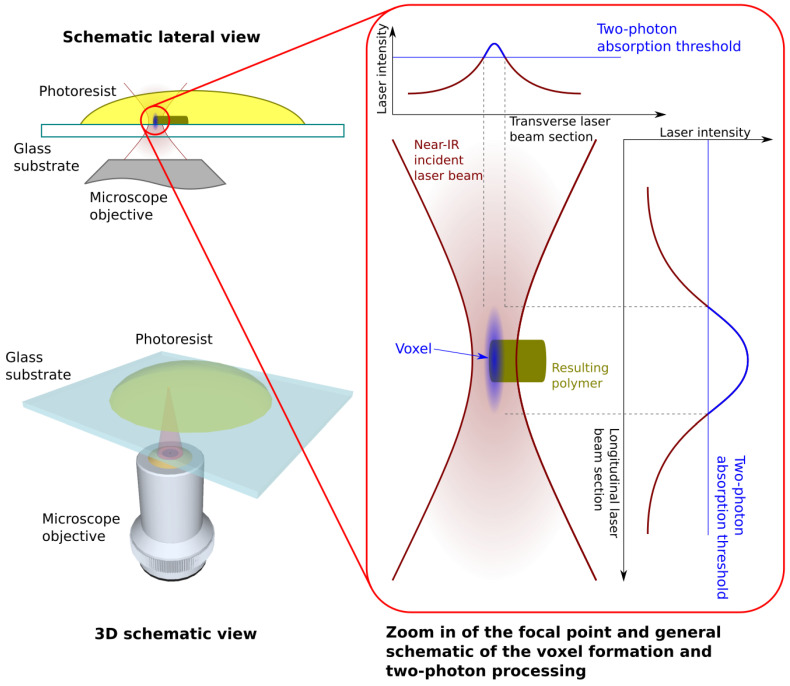
Working principle of Laser-Direct Writing via Two-Photon Polymerization technology.

Figure 3 illustrates the general working principle of LDW via TPP technology. Infrared ultrashort laser pulses are strongly focused inside a photopolymerizable resin. The two-photon absorption threshold of the resin is overcome in a small prolate volume, named “volume pixel”, where a chain polymerization process is initiated.

LDW via TPP allows the fabrication of highly complex free-form 3D micro/nanostructures, of high interest for scaffolding in tissue engineering and regenerative medicine [21,29,85]. Such structures known as scaffolds can be used for growing artificial living tissue which can be then implanted and integrated into a host natural tissue. By selecting the appropriate biomaterials for structures’ fabrication, LDW via TPP can be used for high-resolution and high-precision fabrication of 3D scaffolds, which allows for high reproducibility of extracellular matrices encountered in the living tissues [21,29,84,85]. Moreover, the near-IR incident wavelengths used in LDW via TPP do not induce cytotoxicity and can, therefore, be used for cells containing and manipulation. Studies on cell exposure to IR wavelengths have demonstrated that they have an impact only on cancerous cells, whereas the viability of normal cells has not been affected by the exposures [86,87,88].

## 4. Actuation of 3D Micro/Nanostructures Using Different Types of Stimuli, for Tissue Engineering Applications

A way to control the cellular behavior in order to obtain new functional tissues is by seeding the cells onto 3D micro/nanostructures called scaffolds, where they attach, interconnect, proliferate and start to form the new desired tissue [89]. A critical condition is that the 3D micro/nanostructures accurately mimic the composition and architecture of the natural tissues [22,89,90,91,92,93]. Cell adhesion to specific surfaces is influenced by both surface chemistry and surface topography [94]. Among the important properties of the 3D micro/nanostructures are morphology, chemistry and cell adhesion properties. There are also several other properties that can be conferred to the structures, such as electrical conductivity or magnetic properties [89,90,91,92,93,94]. To date, 3D micro/nanostructures have been stimulated through exposure to mechanical, chemical, electric and magnetic stimuli ([23] and references therein).

In the last decades, different combinations between 3D architectures and other properties of the structures, such as morphology and surface chemistry, provided the means to tailor the structures in a manner that they reach specific purposes such as the recruitment and differentiation of specific cell types [95]. For example, structures’ coating with collagen–chitosan components was found to change both the chemistry and the morphology of the structures, which further impacted the attachment and osteogenic differentiation of bone-forming cells [21]. Aside from morphology and chemistry, additional properties of the 3D structures have been also addressed. For example, electrically and biocompatible conductive polymers such as polypyrrole allowed the use of 3D structures as electrically conductive reservoirs for the controlled delivery of drugs [30]. Moreover, the fabrication of magnetically active 3D structures from photosensitive polymers with embedded magnetic nanoparticles promoted the osseointegration of the structures seeded with osteoblast-like cells and fastened the bone regeneration process; more specifically, when exposed to static magnetic fields, the cell-seeded structures accelerated the proliferation and differentiation of osteoblast-like cells [96]. 

### 4.1. 3D Structures Actuated through MicroTopographies

One of the most important disadvantages of existing 3D structures that act as scaffolds for tissue engineering is that most of the cells quickly adhere to the outer part of the structure, obstructing the permeation of cells inside the whole volume of the structure, which leads to the formation of a necrotic core [86]. Moreover, these structures have a random spatial configuration that cannot be reproduced and cannot maintain the volumetric isotropy characteristic for the majority of the living tissues. For example, bone tissue has a hierarchic structure, and therefore, whenever a transplant is developed for replacing missing or afflicted tissue, it is necessary to understand how each section of the 3D micro/nanostructure fabricated to act as scaffold is formed and what tasks it fulfills in the native tissue [86].

Until recently, complex multi-scale 3D structures were designed using top-down, bottom-up and hybrid approaches. The top-down approaches, such as nano-printing lithography, soft lithography and capillary force lithography, require additional efforts such as specific pressure application, the delivery of specific heat quantities or covering the substrate surface with a thin adhesive film that exceeds the printing matrix adherence to the modeled substrate ([94] and references therein). The bottom-up methods, such as self-assembly techniques, have been used to fabricate complex 3D biomimetic structures, but they showed poor precision because of uncontrollable parameters such as chemical and physical states of the surface (including defects) ([94] and references therein). Presently, an important challenge is positioning biomaterials in specific places on microstructures using hybrid methods which combine top-down and bottom-up approaches, such as pre-modeling, post-structuring or pre-shielding using shielding matrices and post-structuring.

Recently, interest in tissue engineering has significantly increased in both academia and the industry. Relentless attempts for finding new methods of replacing afflicted organ tissue/organ parts started from the desire of not only improving the quality of life, but also prolonging it. Current trends concentrate on producing structures that are similar to the architecture and composition of tissues, or even natural organs, whose architecture is similar to native tissues. To attain such an objective, it is necessary to create implantable 3D structures that are non-toxic and whose 3D architecture allows for cells to adhere, grow, interconnect and differentiate until they reach the state of functional tissue [21,22]. 

The major challenge in tissue engineering is the replication of complex 3D structures encountered in nature, at micrometric and nanometric scales, so that they closely reproduce the extracellular matrix ECM architectures from in vivo cellular environments. The majority of the natural tissues possess hierarchal architectures based on fibrillar and/or tubular unitary elements [95]. The variations of the biophysical properties, and thus of the functionality of these structures, are given by the variations in size, spatial arrangement and chemical composition of different elementary elements. Another important aspect of engineering functional tissues is the structural and functional anisotropy of the tissue. The approaches to fabricating 3D structures similar to the in vivo environments are extremely broad and, therefore, a complete overview on such a topic is almost impossible to be made. Among the wide pool of technological approaches, materials and architectures, one could mention for example the fabrication of arrays of microchannels by a CO_2_ laser engraving system within alginate macroporous scaffolds, for obtaining a blood-vessel-supporting microenvironment [97,98] have used soft lithography technique to generate network-like tissue patches composed of cardiomyocytes, where the cells and secreted ECM proteins alignment was improved by extending the transverse diameter of the elliptical pores that crossed the patch networks. A laser-foaming technique has been employed to fabricate an array of microscale porous polylactic acid scaffolds for tissue-based biomedical assays, which demonstrated enhanced cell viability within the scaffolds [96].

It has been proven that cell adhesion to a surface is strongly influenced by surface topography [22,90,91]. In this context, the major challenge is to obtain microstructures that are reproducible, complex and capable of controlling cell adhesion. From one side, some applications require the microtopographies of the 3D structures to minimize the interaction between specific cell types and structure, which is highly important for developing new devices, such as biosensors, blood-interacting devices and anti-microbial surfaces, where microorganism adhesion, such as cells or even bacteria, could limit the functionality of the device [99,100,101]. On the other side, there are specific applications that require increased cell adhesion on the 3D micro/nanostructures [94]. Each aspect can be controlled through specific microtopographies. For example, it has been shown that endothelial, osteoblast, phenotype neural cells and stem cells respond differently to topographies that contain microscale elements. It has been found that the width, spacing and depth of these topographical elements have a major impact on cell behavior. As such, in order to control cell adhesion, a broad variety of microarchitectures has been fabricated, in the shape of as channels, pillars and cones [85].

**Figure 4:**
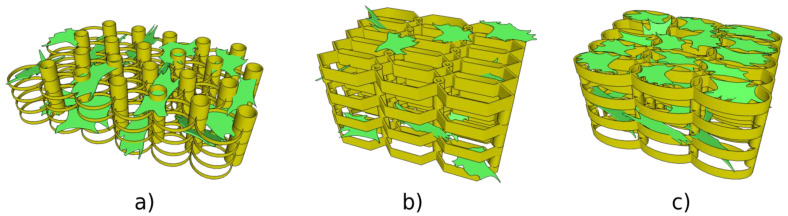
Three-dimensional scaffolds for bone-tissue engineering fabricated using LDW via TPP, (**a**) high porosity, (**b**) improved volumetric cell density, (**c**) optimized volumetric cell density and adhesion.

Figure 4 illustrates several designs of three-dimensional scaffolds developed to address specific objectives for bone-tissue engineering, i.e., high porosity, cell density and cell adhesion, with the purpose of obtaining a geometry optimized for high cellular density, adhesion and viability.

In this context, Laser-Direct Writing via Two-Photon Polymerization (LDW via TPP) has been recently used for generating micrometric 3D structures that were capable of guiding/controlling cell adhesion [23]. Surface engineering at a micrometric scale can significantly improve the structures’ performances, given the known fact that the structure’s microtopography impacts structures wettability, mechanical resilience and adhesion properties. To sum up, the microtopography of the 3D structures is of utmost importance for biomedical applications, such as implantable scaffold-like structures for tissue engineering, because the 3D microstructures go directly into contact with the biological fluids and the cells and tissues in the vicinity of the implant [94].

In recent years, we have reported a series of microscale 3D scaffolds fabricated using LDW via TPP [21,23]. These microstructures have an iteratively optimized geometry for improved osteoblast-like cell adhesion, permeation and proliferation. The fabrication method allows for high reproducibility and can maintain volumetric isotropy. In order to determine an optimal geometry, osteoblast-like cells have been seeded on various 3D geometries and their behavior analyzed. Recent studies show how pore size and density influence cell behavior [102,103,104,105]. 

Recently, LDW via TPP was successfully used for the fabrication of optimized 3D honeycomb-like microstructures that were seeded MG-63 osteoblast-like cells [21]. The design addressed several recurring issues in tissue engineering: the formation of a necrotic core due to cells rapidly attaching on the outer edges of the structure and restricting cell and nutrient in-volume penetration, fabrication reproducibility, geometric isotropy and control. The possibility of controlling 3D spatial cell growth by adjusting the geometry and porosity was demonstrated (Figure 4). For layer separation between 2 μm and 10 μm, cells have been shown to gradually penetrate the scaffolds. Moreover, the microstructures have been shown to induce stronger osteogenic differentiation (1.5 times higher ALP activity), mineralization (1.3 times higher amount of calcified minerals) and osteocalcin secretion (2.3 times higher) in comparison to other structures. Furthermore, scaffolds with separation either below 2 μm or above 10 μm exhibited poor mineralization ability and were not able to make interconnections. 

**Figure 5:**
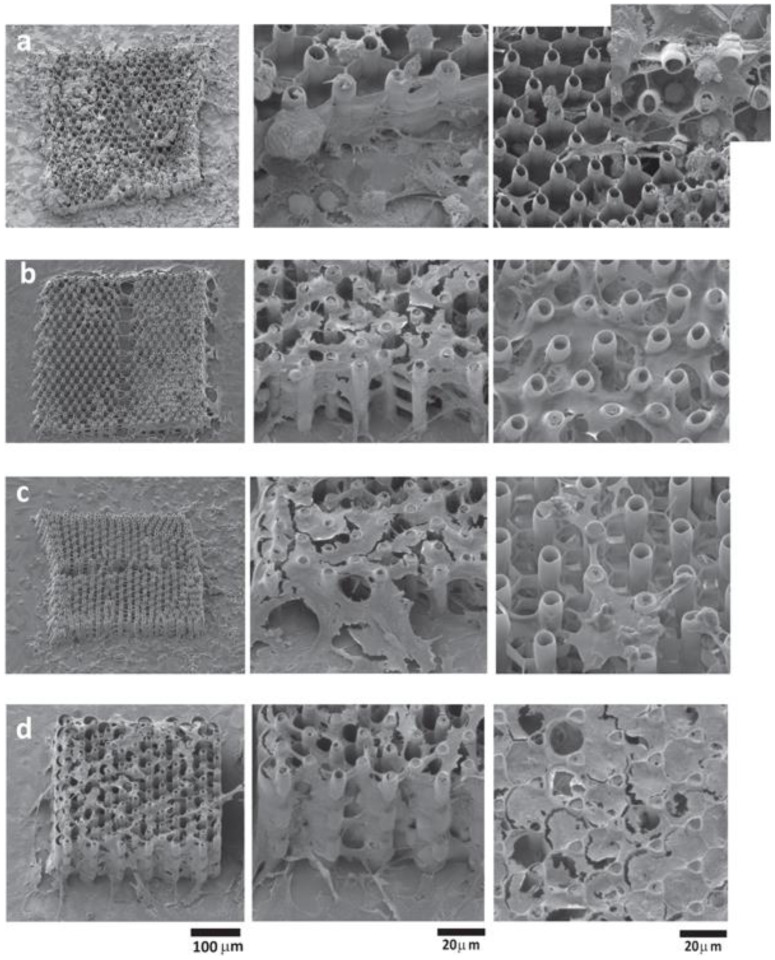
Scanning electron micrographs of MG-63 osteoblast-like cells seeded for 72 h on structures with heights of the circular elements/layers’ separations of: (**a**) 2 µm/2 µm; (**b**) 2 µm/10 µm; (**c**) 2 µm/20 µm; (**d**) 15 µm/10 µm. Figure reproduced from reference [21]: Paun et al. “Laser-direct writing by two-photon polymerization of 3D honeycomb-like structures for bone regeneration”. ©IOP Publishing. Reproduced with permission [21]. All rights reserved.

Figure 5 shows the cells penetrating inside 3D multilayered structures having circular elementary units separated by vertical pillars, where the heights of the circular elements and the separation of the layers varied according to the figure legend. The scanning electron microscopy images from Figure 5 indicate that the separation of the layers and the heights of the circular elements both influenced the cell attachment in and into the structures [21].

**Figure 6:**
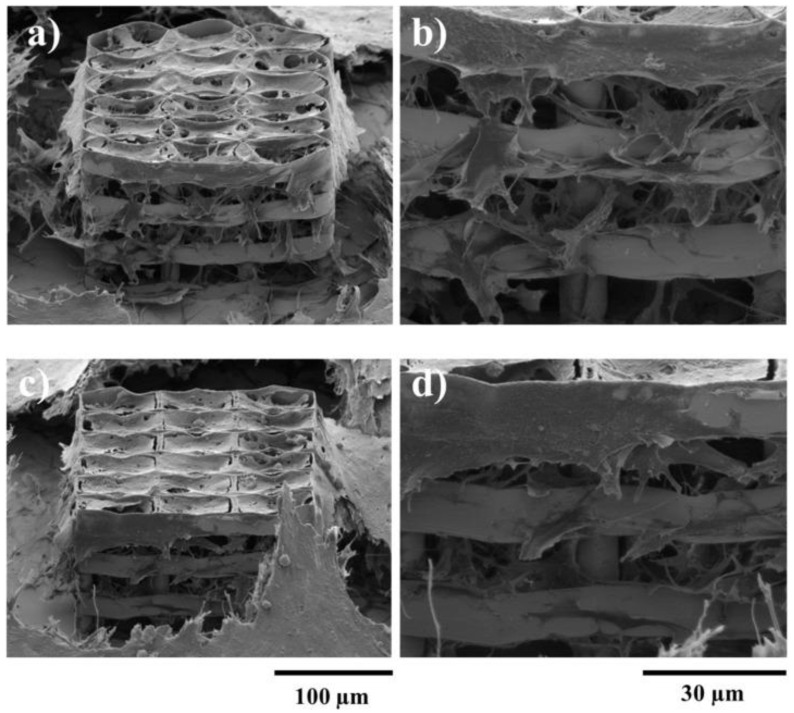
Scanning electron micrographs of MG-63 osteoblast-like cells growing on ellipsoidal (upper panel) and hexagonal (lower panel) multilayered 3D structures having the layers spatially separated by cylindrical pillars, after 7 days in cell culture. (**a**,**c**) Overviews; (**b**,**d**) Closer, tilted side view, showing cells penetrating inside the structure. Reproduced with permission [23].

In addition, the geometry of the elementary units of the structures was found to be equally important for controlling cellular attachment. In this regard, structures having ellipsoidal and, respectively, hexagonal elementary units were compared [101], the structures that allowed the most uniform cell distribution throughout the whole volume of the structures were the ones having ellipsoidal elementary units (as it was schematically illustrated in Figure 4b,c and experimentally proved by the scanning electron micrographs from Figure 6).

LDW via TPP has been also recently used for the fabrication of multilayered microstructures with elliptical and hexagonal units, arranged in several layers separated by microtubes; these structures allowed the cell attachment, permeation, proliferation and differentiation [101]. It was demonstrated that an appropriate structure, i.e., over 85% porosity and a layered design of the 3D structures allowed for mass transport inside the volume of the microstructures, which in turn, improved the cell viability and proliferation also indicated by studies reported by Mohanti et al. [106]. The cells seeded on 3D microstructures with optimized geometries, i.e., having a multilayered architecture based on unitary elements in the shape of ellipses separated by vertical microtubes, induced a 3.5 times increase in Alkaline Phosphatase production and a 2.3 times increase in osteocalcin secretion when compared to the control samples, i.e., flat polymeric substrates, indicative of the fact that the 3D microstructures with optimized microtopographies increased and fastened the cells differentiation and mineralization towards the formation of bone tissue [101].

### 4.2. 3D Structures Actuated through Hierarchic Micro/Nano-Topographies

Another strategy for enhancing the functionality of 3D micro/nanostructures for tissue engineering applications consists of the fabrication of 3D structures having different levels of hierarchy, namely structures that contain sub-structures with different size scales, ranging from micrometers to nanometers [85]. Particularly effective for controlling cellular behavior are hierarchical structures that contain elements with overlapping length scales [24,107]. Endothelial cells, osteoblasts, neural phenotypic cells and stem cells, among other cell types, have been demonstrated to respond differently to hierarchical topographies made up of nano- and microscale structures [24,108].

Nature shows that the most efficient constructs for promoting various functions of different cell types are the hierarchical structures, i.e., structures that combine multiple size scales that act in parallel [109]. Current strategies for the fabrication of hierarchical biomimetic structures address different levels of tissue organization, starting with the development of molecular templates for in vitro tissue regeneration at the nanoscale, followed by the fabrication of biomimetic scaffolds having 3D architectures at the micro- and nanoscale, and, finally, the application of external stimuli at the macroscale, to enhance/fasten the tissue growth within the biomimetic scaffolds [110].

To date, hierarchical structures have been obtained by top-down, bottom-up and hybrid, i.e., top-down combined with bottom-up approaches [111,112,113]. The top-down methods such as nanoimprint lithography, soft lithography and capillary force lithography necessitate multiple processing steps such as applying pressure, heat or coating the surface of the substrate with a thin adhesive layer that overwhelms the adhesion between the imprint mold and the patterned layer [24,106]. The bottom-up methods (for example, self-assembly) were able to produce more complex 3D structures, but they were less accurate due to uncontrollable parameters such as surface chemical and physical states (including defects) [108].

Efimenko et al. reported hierarchical wrinkled surface topologies with millimeter-scale waves donned with controllable periodic surface structures ranging from ~50 nm up to 500 μm, albeit obtained using a multistep stochastic fabrication process, which limits control and reproducibility [24]. The purpose of the study was to develop cell-repellant surfaces for marine anti-fouling applications. A noticeable difference between the control and the dual-scale surface was the growth of barnacles, i.e., control samples (flat surfaces) exhibited barnacle recruitment after 2 months in seawater, while the dual-scale surfaces showed no barnacle recruitment after 16 months of immersion in seawater.

**Figure 7:**
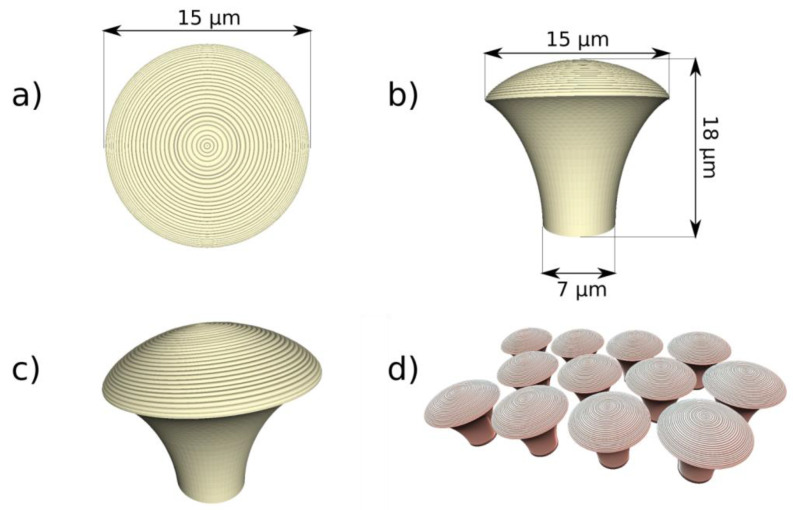
Dual scale cell-repellant microstructures, single element (**a**) top view, (**b**) side view, (**c**) isometric view, (**d**) spatial positioning of several elements (mushroom-like structures).

In a recent study, it was reported the design and fabrication of innovative hierarchical structures with cell-repellency capability, using laser-direct writing via two photons polymerization LDW via TPP [85]. The structures were designed in the shape of “mushrooms”, with an underside (mushroom’s leg) that served as a support structure and a top side (mushroom’s hat) that was decorated with micro- and nanostructures (see Figure 7). On top of the mushrooms, a ripple-like pattern was created with length scales ranging from several micrometers (Microstructured Mushroom-like Pillars, MMP) to tens of nm (Nanostructured Mushroom-like Pillars, NMP). 

**Figure 8:**
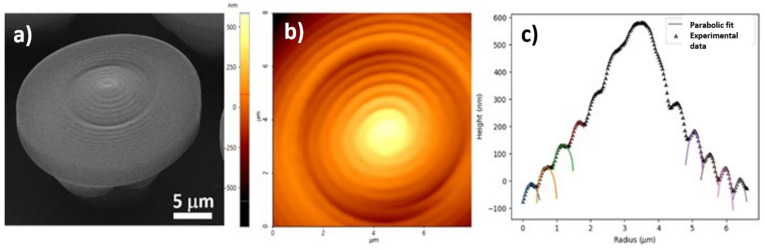
(**a**) Scanning electron micrograph of a nanostructured mushroom-like pillar (NMP) inclined at 30 degrees; (**b**) Atomic force microscopy (AFM) image of NMP from (**a**); (**c**) Cross-section of the indentation surface through the center of a mushroom-like pillar (star-like points: experimental data; continuous lines: parabolic fit). Reproduced with permission [94].

Scanning electron microscopy and atomic force microscopy were used for investigating the optimum structures’ design and laser-processing parameters for LDW via TPP fabrication over the micro- and nanostructures, with sub-micrometric spatial control (Figure 8).

**Figure 9:**
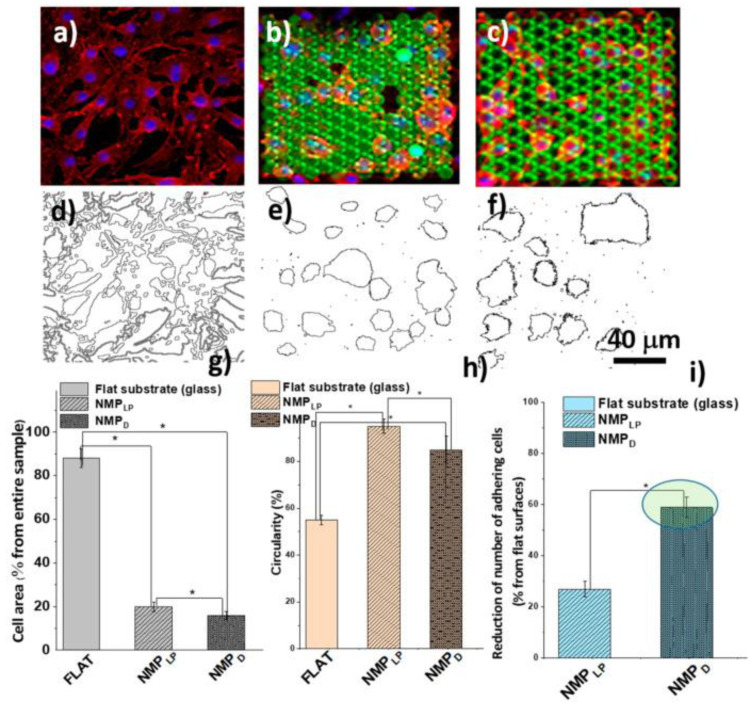
Fluorescent images of cells on: (**a**) glass (control), (**b**) nanostructured mushroom-like pillars with optimized laser power (NMP_LP_), (**c**) nanostructured mushroom-like pillars with optimized design (NMP_D_); green: structures’ autofluorescence; blue: cells nuclei, Hoechst; red: cells cytoskeleton, Phalloidin; (**d**–**f**) image processing by Image J showing only the cells’ outlines; (**g**–**i**) cells area, cells circularity and reduction in adhering cells for flat, NMP_LP_ and NMP_D_ structures, respectively. The scale (40 µm) from (**f**) is valid for all figures, i.e., (**a**–**f**). The sign “*” from (**g**–**i**) indicate that the data are statistically significant. Reproduced with permission [94]. The blue circle in Figure 9i indicates the best result regarding the cell repellency effect of the structures.

In vitro studies were carried out to investigate the cellular response in respect to the hierarchic topographies from Figure 8. As shown in Figure 9, the MMP structures preserved the native cellular shape, namely spindle-like with phyllopodia, whereas the cells from NMP structures had a round shape and no phyllopodia, indicative of cell apoptosis. The cell morphology further impact on the degree of cellular attachment on the hierarchic structures. Namely, the NMP structures decreased cellular adhesion by ~60% as compared to flat surfaces, while the MMP structures were less efficient in impeding cellular adhesion. 

### 4.3. 3D Structures Actuated through Mechanical Stimuli

A current approach in tissue engineering and especially for bone regeneration is based on using mechanical stimulation of 3D structures in order to accelerate cell differentiation [27,114,115,116,117,118,119]. Several methods have been proven to be efficient for this purpose. One of these methods used topological surfaces with appropriate dimensions and rigidity for changing the shape of cell nuclei [27]. The second efficient approach for mechanical actuation of the 3D structures is by Low-Intensity Pulsed Ultrasound Stimulation (LIPUS) [27]. 

Regarding the first approach, i.e., controlling the cell behavior using 3D structures in the shape of topological surfaces with specific microtopographies and stiffness, it focuses on changing the shape of the cellular nucleus in response to mechanical cues from the 3D structures [115,116]. As such, synthetic tissue development has been targeted to the fabrication of topological surfaces, capable of mechanically stimulating the nuclei of bone-forming cells, as an alternative for conventional bone tissue transplant that is always limited by the reduced number of compatible donors [114,115,116,117,118,119]. The cellular nucleus is the largest and most rigid, mechanically sensitive cellular organelle, and plays a crucial role in the regulation of cell mechanics; external forces are transmitted to the cell nucleus through adhesion molecules scattered on the surface of the cells, causing changes in the structure and functions of the nucleus, which in turn affect the mechanical sensitivity of the entire cell [27]. Several attempts have been made in order to fabricate 3D structures in the form of topological surfaces, with high spatial precision and repeatability, able to deform the cell’s nuclei. It was demonstrated that tissue regeneration is mainly improved by nuclei deformation, which in turn is determined by topological surfaces with pillar lattices of appropriate dimensions and rigidity [27]. In this regard, LDW via TPP was used for the fabrication of topographic surfaces in the shape of vertical microtubes with heights between 5 μm and 10 μm positioned in hexagonal lattices with a spacing between the centers of neighboring microtubes of 10 μm. 

These structures were able to deform the nuclei of osteoblast-like cells. ALP activity increased up to 150% due to topology. ALP activity also increased proportionally to microtube heights. LIPUS treatment was found to increase ALP activity by ~50%. Osteocalcin secretion increased ~100% for 20 μm tall microtubes, after LIPUS treatment.

**Figure 10:**
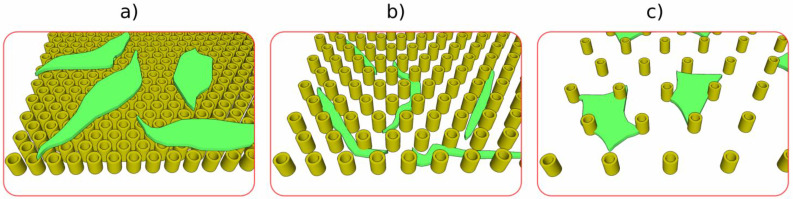
Osteoblast-like cell behavior based on geometry of topological surfaces comprised of 5 μm diameter microtubes, (**a**) tightly packed (<10 μm distance between microtubes), (**b**) averagely packed (>10 μm distance between microtubes), (**c**) loosely packed (>20 μm distance between microtubes). The yellow color correponds to the vertical microtubes fabricated by LDW via TPP and the green color corresponds to the cells seeded on the microtubes.

In all, this approach emerged as a promising method to activate biochemical signaling that improves cell differentiation [119]. Figure 10 illustrates the behavior of osteoblast-like cells on topological surfaces comprised of microtubes positioned in a hexagonal lattice. For distances between tubes of under 10 μm, cells remained on top of the tubes and kept their spindle-like morphology. For microtube separation of above 10 μm (12 μm more precisely, in this case), cells migrated between tubes and modified their shape and size to fit the grooves, i.e., they became elongated and thin, with sizes of down to 8 μm for the minor axis and up to 50 μm for the major axis. For microtube separation of above 20 μm, there were virtually no morphological differences between cells seeded on the topological surfaces and the control (cells seeded on a flat glass substrate) [118].

The second approach mentioned above of the actuation of the 3D structures using Low-Intensity Pulsed Ultrasound Stimulation (LIPUS) [27], relies on a fundamental issue of tissue engineering, specifically the acceleration of tissue regeneration through the mechanical stimulation of cells seeded on 3D structures using appropriate, intensity and duration of the applied stimuli, in order to obtain an increase in cellular metabolism and phenotype adaptability [27]. The fact that cells detect the mechanical impact coming from the environment and translate biophysical and biochemical stimuli to intracellular signals represents an efficient strategy for this purpose [25,26,27,114,115,116,117,118,119]. In order to stimulate mechanically various types of cells, 3D microstructures have been fabricated generally in the form of vertically aligned pillars [25,26]. For this type of structure, it has been demonstrated that Low-Intensity Pulse Ultrasound Stimulation of the seeded cells is a non-invasive and simple-to-use therapy, which was approved by the FDA (Food and Drug Administration, USA) in 1994, for bone fracture treatment [25,26].

**Figure 11:**
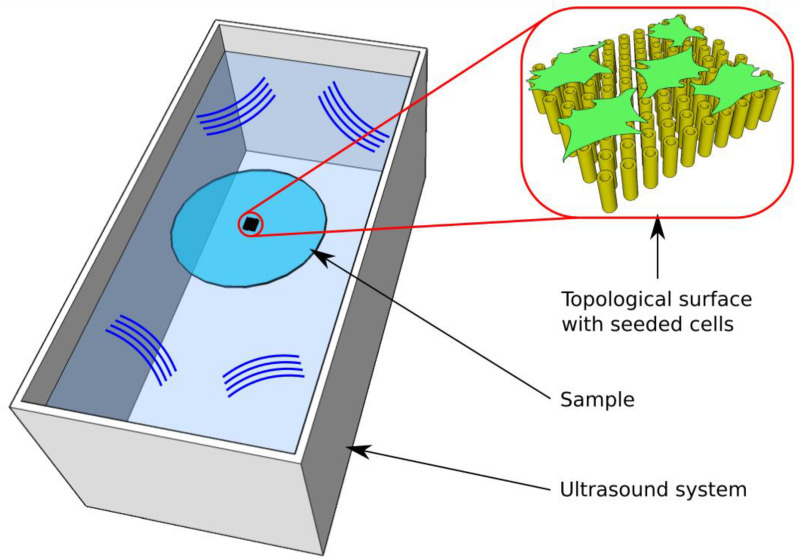
Schematic illustration of the experimental setup used for low-intensity pulsed ultrasound stimulation of topological surfaces comprising of flexible vertical microtubes fabricated by LDW via TPP.

The mechanical stimulation of various cell types has been realized using microstructured surfaces that generally consisted of pillar-like elements arranged in various geometries [120,121,122,123,124]. Recently, it was demonstrated that a proof-of-concept, in which we validated a synergistic effect between topological surfaces fabricated using LDW via TPP and Low-Intensity Pulsed Ultrasound Stimulation (LIPUS), significantly improved the osteogenic differentiation of cells [27]. Figure 11 illustrates a schematic representation of the LIPUS treatment of topological surfaces comprised of vertical microtubes positioned in a hexagonal lattice. The microtube arrays were designed with various heights in order to obtain different flexibilities, and were disposed of in a hexagonal lattice of appropriate geometry so as to allow for the analysis of individual cells [27]. 

**Figure 12:**
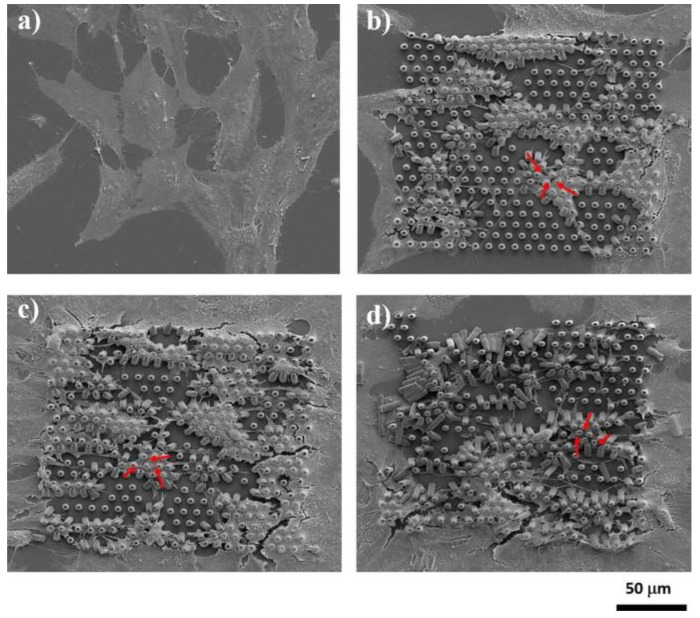
Scanning electron micrographs of MG-63 osteoblast-like cells cultivated for 48 h on: (**a**) flat and microtubes arrays with microtubes heights of (**b**) 5 µm; (**c**) 10 µm; and (**d**) 20 µm. Reproduced with permission from Springer Nature, Journal of Materials Sciences, Osteogenic cells differentiation on topological surfaces under ultrasound stimulation, Paun et al. J Mater Sci 54 11213–11230 (2019). Copyright ©2022. The red arrows indicates the bending of the microtubes unde the action of the traction forces exerted by the seeded cells.

Figure 12 illustrates how LIPUS stimulation of the cell-seeded microtubes arrays induced a certain deformation/bending of the microtubes, which increased with increasing microtubes heights’. These topological surfaces induced significant cell nuclei deformation, even without mechanical stimulation. LIPUS further increased the nuclei deformation effect, which resulted in a 200% increase in the production of osteogenic markers, as determined from measurements of the alkaline phosphatase activity and osteocalcin production [27].

### 4.4. 3D Structures Actuated through Chemical Stimuli

Several 3D printing technologies are used for biomedical applications, yet LDW via TPP offers the appropriate fabrication characteristics for 3D structures that can act as extracellular matrices which aid tissue regeneration. In this sense, however, designing an optimal structure and choosing an appropriate photopolymer is essential. For biomedical applications, a photopolymer must allow for fast processing and a high degree of polymerization, but equally important is that the photopolymer has appropriate mechanical characteristics, chemical and physical stability, as well as good biocompatibility (non-citotoxicity) for cell seeding [29].

Notwithstanding the flexibility in the design and fabrication of practically any architecture, the use of printing technologies for obtaining 3D structures for tissue engineering is very much limited by the available photoresists. For example, methacrylates have been efficiently used in dental applications, while bio-compatible photopolymers derived from biological polymers, such as chitosan and gelatin, have been created for soft tissue regeneration [125,126,127]. It has been proven that IP-L class photopolymers, developed by Nanoscribe GmbH, show good biocompatibility for in vitro bone-forming cells, with promising in vivo results. IP-L 780 photopolymer is a biocompatible liquid formula, optimized for LDW via TPP, with increased light sensitivity for rapid prototyping of 3D structures [21]. However, for optimum tissue formation from the cell-seeded structures (scaffolds), the 3D imprinted structures should be provided with additional properties that promote the cells to grow into functional tissue, among which the chemical composition is one of the most factors to be accounted [84]. Despite the increased scientific interest in the matter, to date the possibilities for the functionalization of 3D microstructures are limited. A recent study showed the deposition capabilities of Initiated Chemical Vapor Deposition (iCVD) to cover 3D-printed structures with polylactic acid and acrylonitrile butadiene styrene combined with hydrophilic polymers such as poly(1H, 1H, 2H, 2H, 2H)-perfluorodecyl acrylate (PPFDA) and glycol-co-diacrylate ethylene glycol poly-methacrylate (P(HEMA-co-EGDA)) [28]. This method has shown several significant disadvantages, however, some of them being directly related to tissue engineering applications. One disadvantage is that iCVD and subsequent deposition techniques required highly complex equipment and multistep experimental procedures. For example, the imprinted 3D structures reached temperatures of up to 80 degrees Celsius during the deposition process, which affected the structure’s mechanical integrity. Moreover, the thermal insulation properties of the 3D imprinted polymeric structures represented an issue for the iCVD process because of high-temperature gradients within the entire structure during processing, which negatively impacted the homogeneity of the deposition.

Coating the 3D structures with natural materials represents a promising alternative to existing functionalization approaches. Natural materials such as chitosan and collagen, are not toxic and promote cell adhesion and proliferation, and therefore offer significant benefits [128,129,130,131]. For example, chitosan has been successfully used in bone tissue engineering due to its high mechanical endurance, low degradation rate and cell-supporting capabilities. These capabilities of chitosan for improving the in vitro adherence of proliferation of osteogenic cells have been proven [128,129,130]. Another promising material for tissue engineering is collagen. For example, for bone regeneration purposes, an increase in osteogenesis gene expression and alkaline phosphatase production was observed in bone marrow cells seeded on structures functionalized with collagen [131]. However, materials such as chitosan (Chi) and collagen (Col) are difficult to process especially because of their weak mechanical resistance; also, it has been found that they might not be appropriate for the management of infected areas. 

**Figure 13:**
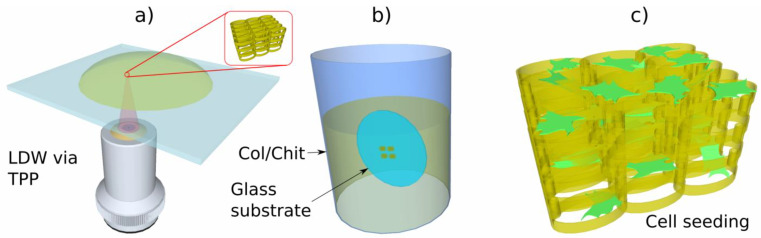
General fabrications steps for Col/Chit coated 3D structures (**a**) laser-direct writing fabrication of the scaffolds, (**b**) Col/Chit coating by structures’ immersion in Col/Chi solution, (**c**) cells seeded on col/Chi functionalized structures.

Considering the above, an innovative approach has been reported in [29], where the mechanical properties of the photocurable material IP-L 780, a biocompatible liquid photopolymer developed by Nanoscribe GmbH and is optimized for 3D microstructuring, with the unique properties of natural materials like chitosan and collagen. The 3D scaffold was fabricated using IP-L 780 for its appropriate mechanical properties, so as to act as a mechanically resistant backbone for the seeded cells. Then, IP-L780 3D microstructures were coated with collagen–chitosan blends of various ratios through a simple immersive process. The concept of this approach is illustrated in Figure 13. The scope was to investigate the influence of the collagen–chitosan ratio (Col/Chi) over the biocompatibility and the osteogenic potential of Col/Chi functionalized (coated) 3D structures against osteoblast-like cells seeded on the structures. The osteogenic effect of Col/Chi-coated 3D structures was much higher as compared to uncoated (non-functionalized) structures, as confirmed by the expression of osteogenic markers such as osteocalcin secretion and alkaline phosphatase [29]. Moreover, the osteogenic effect of the 3D structures actuated by Col/Chit functionalization was the most prominent for a collagen–chitosan blend ratio of 20/80, where the osteocalcin secretion increased ~6.5 times (~1.2 ng/10^5^ cells on control versus ~8 ng/10^5^ cells) [29].

**Figure 14:**
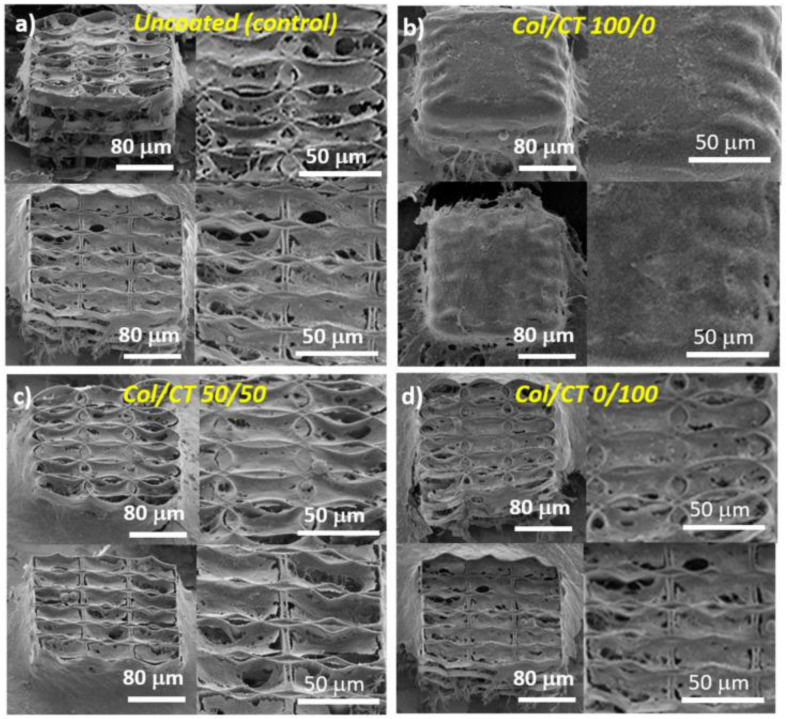
Scanning electron micrographs of MG-63 osteoblast-like cells seeded for 3 days on: (**a**) uncoated 3D structures; (**b**–**d**): 3D structures functionalized with Collagen/Chitosan (Col/CT) with different blending ratios. For (**a**–**d**): the upper panels illustrate cells seeded on structures with ellipsoidal units; the lower panels illustrate cells seeded on structures with hexagonal units; the left panels show overviews of cell-seeded structures tilted with 30°; the right panels show closer views of cell-seeded structures depicted in the left panels. Reproduced with permission [29].

Despite the promising results, the major problem of heterogeneity of the coating remains unresolved, since immersing a complex 3D structure into Col/Chi liquid formulations does not guarantee a uniform coated into the entire volume of the structure. At present, intensive research is being conducted in order to determine the optimal techniques for functionalizing complex 3D structures made of synthetic materials with natural compounds, in order to provide an optimum chemical functionalization of 3D structures, for tissue engineering applications as indicated by reference [29] and the references therein. The role of the coating in terms of Col/CT blending ratio from [29] is revealed in Figure 14, showing scanning electron micrographs of MG-63 osteoblast-like cells seeded for 3 days 3D structures fabricated by LDW via TPP of IP-L780 photopolymer and functionalized/coated with Collagen/Chitosan (Col/CT) with different blending ratios. 

### 4.5. 3D Structures Actuated by Electric Fields

One of the most pressing issues concerning 3D structures acting as scaffolds for tissue regeneration is represented by post-implantation infections [30,131,132,133,134,135,136]. These are caused by inflammatory cells that invade the contact area between the tissue and the implanted scaffold and produce inflammation, fibrosis and, finally, the destruction of the surrounding tissue [131]. 

For this reason, at present, significant efforts are dedicated to the development of therapeutic approaches that reduce chronic inflammation at the site where the scaffolds are implanted. In particular, modern drug-delivery methods focus on local drug delivery using carrier materials and structures, whose implantation ensures a high local drug concentration and minimizes the risk of systemic toxicity, which are characteristic of traditional approaches, i.e., oral drug administration routes [30].

The most frequently used pharmacological agent that inhibits the inflammatory processes by decreasing the immunologic response of the organism is the synthetic glucocorticoid Dexamethasone (Dex) [30,130,131].

The most advanced tissue regeneration techniques are focused on 3D structures that simultaneously promote cellular differentiation and diminish inflammation and post-implant infection [30]. For this purpose, the structures are enriched with controlled drug-delivery systems that allow for the drug to be loaded on the structure’s surface, that acts as an intelligent bio-interface, and then released on demand, under the action of specific stimuli [30]. Furthermore, these controlled drug-delivery systems have been found to increase the drug-targeting specificity, lower drugs’ toxicity, improve the absorption rates and protect the delivered pharmaceutical compound from biochemical degradation [30].

In order to initiate drug release from scaffolds enriched with such controlled drug-delivery systems, UV, visible or near-IR radiation, magnetic fields, ultrasounds and electric stimuli have been used [30,130,131,132,133,134,135,136] with more or less success. Among these, electric stimulation has drawn particular attention [30,129,130,131,132,133,134,135,136]. Moreover, it has been shown that the electric stimulation of cell-seeded structures improves the adhesion, differentiation and proliferation of cells from the nervous system, skin, bone and muscles, which makes it a useful tool for engineering diverse tissue types. Numerous studies have shown the benefits of electric stimulation via conductive substrates, such as metals, graphene and conductive polymers, for bone tissue regeneration [30,129,130,131,132,133,134,135,136]. For example, increasing the translocation of Ca^2+^ through calcium channels controlled by voltages applied to the cellular membrane, as well as Ca^2+^ release from intracellular reserves within the cells, have been correlated with an increased degree of mineralization in electrically stimulated osteoblasts [30].

Although the existing results on electrically controlled drug-delivery systems for tissue engineering are promising, the efficacy of drug loading on the structures’ (scaffolds’) surfaces to combat post-implantation issues such as inflammation of immune rejection must be further improved. To this end, electrically controlled drug-delivery systems have been developed in various shapes, such as micro/nanoparticles, bio-capsules, microneedles and micropumps [129,130,131]. On-demand delivery of drug molecules incorporated in these systems allowed for better control of the dynamics of the drug release, in comparison to conventional static systems, in which delivery rates are established before implanting. 

Regarding the types of materials suitable for fabricating electrically controlled drug-delivery systems for tissue engineering applications, electrically conductive polymers (CP) emerged as the most appropriate materials for this purpose [30,129,130,131,132,133,134,135,136], due to their high strength, good biocompatibility and capability of working at body temperature, as well as in bodily fluids. Polypyrrole (PPy), whose inherent conductivity induces bone tissue formation, is the most frequently utilized conductive polymer, especially for the regeneration of bone tissue [30]. Electric commutation of polymer redox states, as well as the flux of doping ions in and out of the material, dictates the release mechanism of PPy-based systems. Drug molecules are either integrated within PPy films, or delivered through an electrically controlled PPy membrane. PPy membranes have been used to adjust glutamate, dopamine and 5-triphosphate adenosine (ATP) release [132,133]. A membrane with an ionic gate made from a PPy-based film was recently used to control chlorpromazine release [33]. Drug quantities that can be stored and released by the polymer, as well as the range of drugs that can be released, are factors that must be taken into consideration for such applications [30].

Controlled release of penicillin, streptomycin and dexamethasone from Py (penicillin/streptomycin) and PPy (dexamethasone) deposited through electrodeposition on the surface of titanium implants were able to combat the bacterial infection and to lower the inflammation around the implant site [135]. PPy-based drug-delivery systems have been also employed in actuators, such as microfluidic pumps [30].

**Figure 15:**
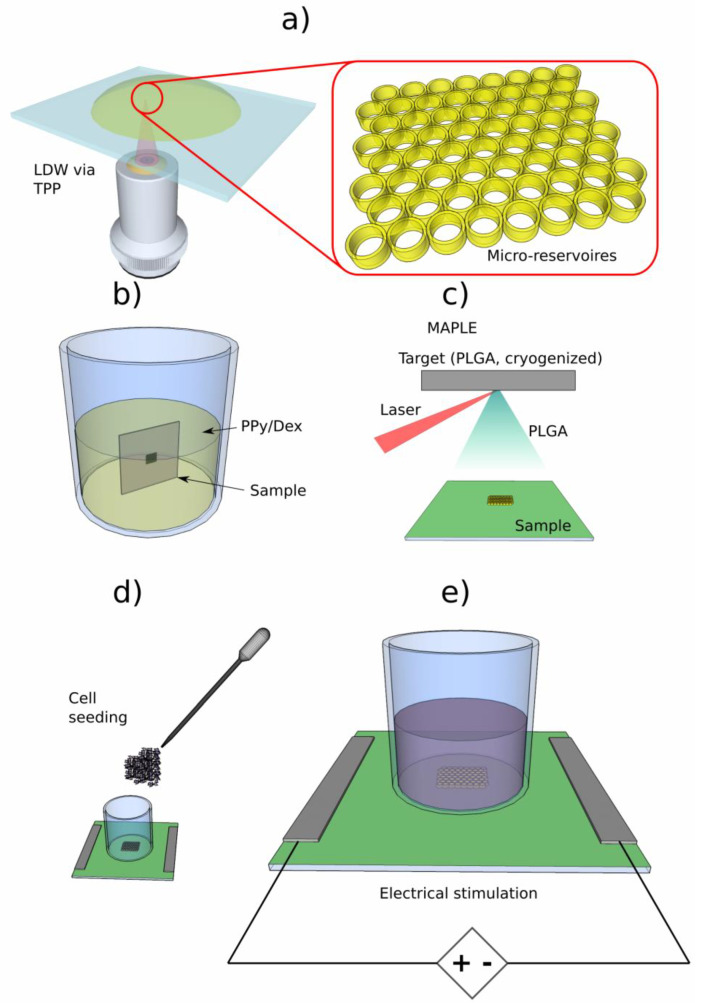
Schematic representation of the experimental steps for electrically activated microreservoirs, (**a**) LDW via TPP fabrication of the microreservoirs, (**b**) filling the microreservoirs with PPy/Dex via immersion, (**c**) sealing the filled microreservoirs with a thin PLGA layer deposited by MAPLE technique, (**d**) cell seeding, (**e**) electrical stimulation for controlled drug release.

**Figure 16:**
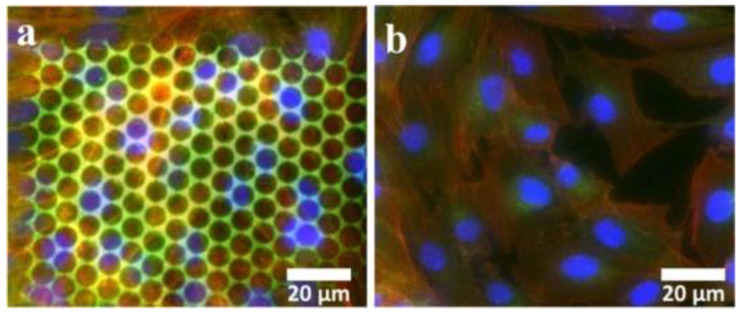
Representative fluorescence microscope images of MG-63 cells grown on: (**a**) Electrically conductive microreservoirs and (**b**) glass, as visualized by immunofluorescence staining of integrin (green: FTIC a protein of focal adhesion plaque; red: F-actin cytoskeleton; blue: cells nuclei). Reproduced with permission [30]: Copyright Elsevier (2022).

We recently developed electrically responsive microreservoirs fabricated by LDW via TPP that released dexamethasone in a controlled manner [30]. PPy was used as the electrically active material. Usually, dexamethasone molecules are either loaded into PPy films, or PPy membranes are used as an electrically active transportation gate [137,138,139]. These mechanisms have some limitations, such as a very low quantity of dexamethasone that can be loaded into those systems. In order to overcome this limitation, we fabricated microreservoirs using the advantages of LDW via TPP to produce (using IP-L780 photopolymer) vertical microtubes of well-determined positioning and volumes. Figure 15 shows the protocol for fabricating the microreservoirs filled with PPy (electrically conductive polymer) and Dexamethasone (Dex, anti-inflammatory drug) and of their actuation via electrical stimuli. The laser-imprinted microtubes were used as microreservoirs that could be easily loaded with a PPy–Dex solution, via a simple immersion process. After draining the sample and drying for 48 h at room temperature, the PPy/Dex from inside the microreservoirs have been sealed with a ~700 nm thick poly(lactic-co-glycolic acid) (PLGA) biodegradable film deposited via MAPLE technique, for limiting the passive (i.e., non-stimulated) drug release that could arrive if the microreservoirs were not sealed. Then, as illustrated in Figure 15, polypropylene tubes were glued around the microreservoirs in order to construct wells for cell seeding. Osteoblast-like cells were seeded onto these structures and the role of electrically controlled Dex release on the cell’s osteogenic potential was assessed. Under electric stimulation, the PPy molecules increased their volume and pushed dexamethasone molecules through the PLGA sealing membrane. As a consequence, the samples that were not electrically stimulated showed a only steady linear release of dexamethasone for the first 120 h, with only 30% of Dex being released at the end of the investigated time interval. Opposing this, the electrically stimulated samples enabled a 98% release of Dex at the end of the stimulation protocol. The osteogenic efficiency of these systems was validated in vitro and showed a 220% increase in alkaline phosphatase activity (indicative of the cell’s osteogenic differentiation) as compared to the unstimulated samples [30]. Fluorescence microscopy images of cells growing on the electrically conductive microreservoirs as compared to cells seeded on a glass flat slide is shown in Figure 16. 

### 4.6. 3D Structures Actuated by Magnetic Fields

A great challenge in tissue engineering is the fact that the tissue regeneration process requires a significant amount of time to obtain a fully functional tissue. The cells are usually seeded ex vivo on 3D structures that are biocompatible and, in common cases biodegradable, where the cells adhere, proliferate and interconnect in 3D networks similar to the architecture of a neutral tissue. The 3D architecture of such structures should allow for adequate colonization of host cells for tissue regeneration after implanting in the affected area [140]. 

Researchers acknowledged since 1996 that the human tissues are at the boundary between diamagnetic and paramagnetic states (susceptibility between ~−11 × 10^−6^ and −7 × 10^−6^), near water susceptibility (−9.05 × 10^−6^ [141]). It is also known that water susceptibility is owed to Langevin diamagnetism, with a small contribution (10%) from van Vleck paramagnetism [141]. As such, it has been proven that magnetic field stimulation accelerates the regeneration of various types of tissues, especially for bone, by helping the scaffold integration by promoting an increased bone density of the newly generated tissue through the increase in calcium content, which further allows for a faster repair of the affected bone [31,140,141].

In particular, static magnetic fields have been found to accelerate the proliferation, migration, orientation and differentiation of osteoblast-like cells, as well as to stimulate the osteogenic differentiation in bone marrow-derived mesenchymal stem cells [31,32,33,34,35,36,37,38,39,40,41,42,101,142,143,144,145,146,147,148,149]. Static magnetic fields, that are externally applied via a magnet positioned in the vicinity of the cell-seeded 3D structure, have been shown to promote in vitro the osteogenesis differentiation of osteoblast-like cells and to trigger the peri-implant bone tissue growth in vivo [32].

Studies have shown that moderate-intensity static magnetic fields, e.g., of about 15 mT promote the osteoblastic proliferation and differentiation of mesenchymal cells by upregulating the genes associated with mineralization and calcium-binding proteins, which enhances the cell mineralization. Although static magnetic fields certainly improve the bone regeneration process, the complete pathways through which they exert their effect on bone formation are not fully understood and further studies are required [32,35]. It is believed that, when a static magnetic field is applied, the magnetic nanoparticles within the scaffolds generate some microdeformations of the structure, which exerts a strain stimulation to the cells seeded into the scaffolds; this strain stimulation then activates the cells to proliferate and differentiate towards the formation of new bone tissue ([101] and references therein). 

The easiest route to fabricate 3D structures with intrinsic magnetic properties; the main approach to confer magnetic properties to the structures, has been to incorporate magnetic nanoparticles (MNPs) into biomaterials [101,140] The best choice was to use superparamagnetic nanoparticles (i.e., these are magnetically activated only when a magnetic field is applied); these MNPs have improved in vitro cell adhesion and differentiation as well as in vivo tissue formation [31]. It was also demonstrated that modifying the magnetic characteristics of the MNPs in the presence of a magnetic field did not have any side effect on cytotoxicity [31]. Moreover, the use of superparamagnetic MNPs has been demonstrated to improve the physico-chemical characteristics of the materials and allows for a more precise replication of the specific hierarchical nanostructure of the bone tissue [33,34]. Furthermore, the metabolism of iron from within MNPs promotes the growth of both bone and non-bone cell series, while also showing a beneficial impact over bone density [140]. Interestingly, the superparamagnetic MNPs have the capacity to promote cell adhesion and development even in the absence of an external magnetic field, due to their intrinsic magnetic properties [30]. Owing to all these significant advantages, to date, composite materials that incorporate MNPs in various matrices proved to be promising in various tissue substitutes. For example, ceramic composites containing superparamagnetic MNPs, hydroxyapatite and tricalcium phosphate have demonstrated good biocompatibility for osteoblast-like cells [36]. The osteogenesis of osteoblast-like cells has been promoted by films based on biodegradable magnetic nanofibers formed by Fe_3_O_4_, chitosan and polyvinyl alcohol, fabricated via electrospinning [101].

Presently, implantable magnetically responsive 3D micro/nanostructures have been realized either by immersing conventional structures in aqueous ferrofluids that contained iron oxide nanoparticles covered with various biopolymers, or through the direct nucleation of the biomimetic phase and superparamagnetic MNPs on collagen fibers by self-assembling [37,101]. Previous attempts of the fabrication of magnetically active structures have been ceramics, gelatin or lyophilization MNPS-saturated polymers, immersion or direct fiber deposition.

Superparamagnetic MNPs focus on the externally applied magnetic field and generate high-gradient magnetic fields within the entire cellular body [38,39]. The intensity of the magnetic forces within the static magnetic fields with gradients of 10^4^ T/m is similar to the gravitational forces and have an effect on cell behavior, in the sense that, for example, the cell migration towards areas of higher magnetic field gradient is favored by such gradients [40,41].

Despite these benefits, until now there are a limited number of studies dedicated to the fabrication of 3D micro/nanostructures that incorporate magnetic nanoparticles for tissue engineering. Presently, the most difficult task is to fabricate magnetic structures with reproducible architectures that contain a precise amount of MNPs homogeneously distributed within the entire volume of the structure [140].

**Figure 17:**
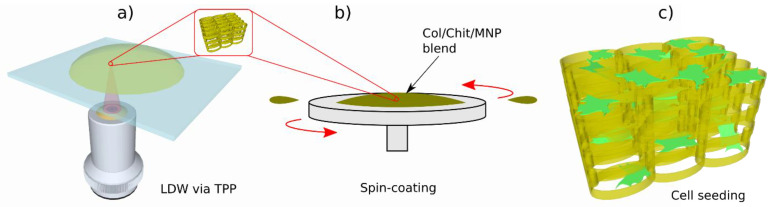
Schematic representation of the fabrication steps for magnetically responsive 3D scaffolds. (**a**) LDW via TPP fabrication of the 3D scaffolds, (**b**) 3D scaffolds coated with Col/Chit/MNP blend, (**c**) cell seeding on Col/Chit/MNPs-coated scaffolds.

In recent works, the issue of magnetic stimulation of cells using 3D magnetically active structures stimulated in static magnetic fields has been addressed in two different ways. One approach is illustrated in Figure 17 and enabled the fabrication of 3D with desired and reproducible geometry using LDW via TPP of IP-L 780 photopolymer, followed by the structure’s coating with a composite made of collagen–chitosan–hydroxyapatite–superparamagnetic MNPs [101]. External static magnetic field stimulation of the functionalized structure was achieved using externally applied static magnetic fields between 100–250 mT. The osteoblast-like cells grown on magnetically stimulated structures presented more than a 200% increase in alkaline phosphatase production, with a proportional increase in the intensity of the magnetic field, reaching an almost 3-fold increase for structures stimulated in a static magnetic field of 250 mT [101].

**Figure 18:**
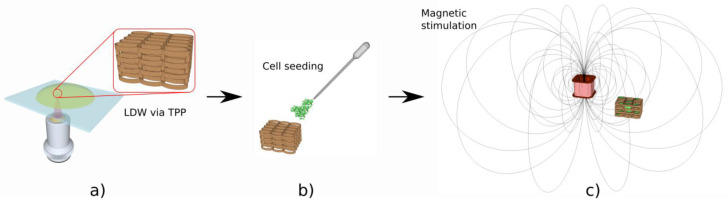
Schematic illustration of the experimental steps for magnetically active 3D scaffolds. (**a**) LDW via TPP fabrication of scaffolds using a composite photopolymerizable material with embedded superparamagnetic nanoparticles, (**b**) cell seeding on the scaffolds, (**c**) static magnetic stimulation of the seeded cells.

The second type of approach was by LDW via TPP fabrication of 3D microstructures that were intrinsically magnetic. This was done through the use of a composite photopolymerizable material made of a blend between a photopolymerizable photopolymer and superparamagnetic magnetic nanoparticles MNPs [150]. The protocol used for this method is schematically illustrated in Figure 18. This approach offered a solution to the difficult challenge of obtaining reproducible magnetic 3D structures with precise, controllable and homogeneously distributed concentrations of the MNPs. We approached this issue by developing a photopolymerizable material with incorporated superparamagnetic nanoparticles that could be processed using LDW via TPP that further enabled us to obtain fully controllable 3D architectures [140]. The ormocore photopolymer was selected owing to its biocompatibility, physical properties and suitability for bone tissue engineering. Ormocore has been previously used for magnetically responsive composite materials, but usually in combination with other techniques [151,152,153]. In the experiment, MNPs density of 4 mg/mL was identified as the best for obtaining mechanically stable structures. 

**Figure 19:**
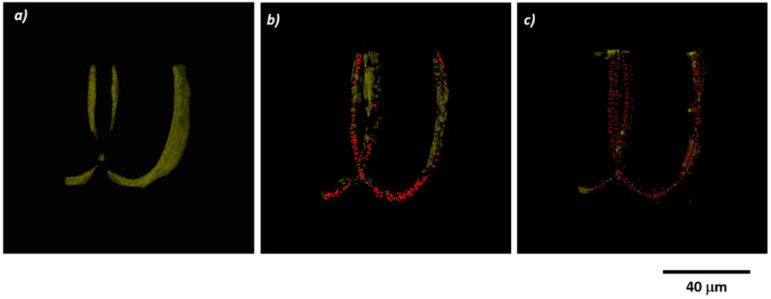
Images obtained by enhanced dark-field microscopy using the Cytoviva three-dimensional (3D) module, for scaffolds with embedded magnetic nanoparticles concentrations of: (**a**) 0 mg/mL; (**b**) 2 mg/mL; (**c**) 4 mg/mL Reproduced with permission [140].

The presence of the MNPs within the whole volume of the structures was demonstrated by enhanced dark-field microscopy (Figure 19) [140].

**Figure 20:**
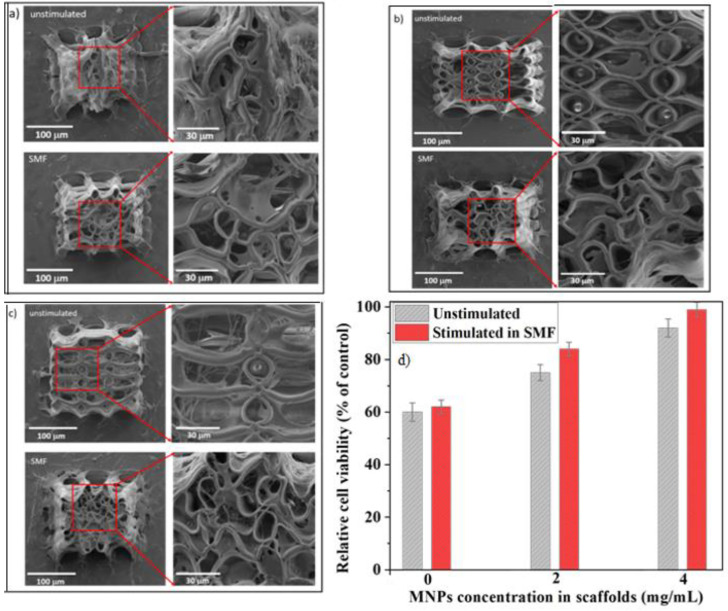
Scanning electron micrographs illustrating cells attached on scaffolds with magnetic nanoparticles concentrations of: (**a**) 0 mg/mL; (**b**) 2 mg/mL; (**c**) 4 mg/mL, after 3 days of cultivation, in the absence (upper panel) and in the presence (lower panel) of static magnetic field (SMF). Left panels: cells growing on the scaffolds. Right panels: insets; (**d**) relative cell viability as a function of magnetic nanoparticles concentration in the scaffolds; except for 0 mg/mL concentration, the results were statistically significant (*p* < 0.05). Reproduced with permission [140].

Osteoblast-like cells were seeded on the magnetically active structures and exposed to a static magnetic field of 1.3 T. The effects of the externally static magnetic fields on seeded osteoblast-like cells have been investigated in comparison with on non-magnetic structures (i.e., fabricated by the photopolymer alone). In the absence of magnetic field stimulation, in all structures, the cells showed a mature osteoblast phenotype, but the number of attached cells increased proportionally to MNPs concentration. An important result was that the stability of the 3D structures increased with MNPs concentration. One consequence was that the mechanical deformation of the cell-seeded structures increased with decreasing MNPs concentration. During SMF stimulation, the cells deformed the 3D structures at a higher extension than the non-stimulated samples (Figure 20). These results could be attributed to the fact that the presence of nanoparticles increases the surface roughness of the structures, therefore providing more surface area for cells to attach. When the static magnetic field was applied, the number of attached cells significantly increased, being proportional to the MNPs concentration. As such, it was possible to fabricate 3D superparamagnetic scaffolds with a homogeneous distribution of MNPs and arbitrary geometry provided by LDW via the TPP technique [140]. Alizarin Red Staining fluorescence intensity increased with MNPs concentration, up to 240% for a MNPs concentration of 4 mg/mL, indicating an increase in the mineral deposits. Samples exposed to SMF have shown a further increase in mineral deposits of up to 50% for the 4 mg/mL MNPs concentration.

## 5. Conclusions

In this review, we provide an overview of the laser-based fabrication of 3D microstructures designed for tissue engineering applications, as well as their actuation using different types of stimuli (topographic, mechanical, chemical, electric and magnetic), with the aim of improving the cellular response. The most commonly encountered and generally effective laser-based fabrication methods are presented and discussed. Further, we emphasize the concept and characteristics of Laser-direct Writing via Two-Photon Polymerization (LDW via TPP) technique for the fabrication of 3D structures for tissue engineering applications.

Regarding the structures, we first address their role as backbones for the cells to attach, migrate, proliferate and differentiate towards the formation of functional tissue. One of the main issues of existing 3D structures for tissue engineering is the fact that cells quickly attach to the surface of the structure, impeding the volumetric migration of the cells and thus, their access to nutrients, which in turn result in low cellular densities and the formation of necrotic cores. Another issue is that, even though there are several approaches regarding the fabrication of 3D structures for tissue engineering, only a few can offer appropriate reproducibility and control of the achievable architectures as well as a good biocompatibility against the seeded cells. LDW via the TPP technique stands out as it offers excellent control over the geometric features at micro- and nanoscales, as well as the possibility to employ biocompatible materials for building the structures.

Furthermore, we address the fabrication and characteristics of stimuli-responsive structures, with an emphasis on the synergy between the geometry and various stimuli that work together for improving the cellular processes and, thus, tissue regeneration. In particular, we address topological, mechanical, chemical, electric and magnetic stimulation.

Topological stimulation relies on the geometric characteristics of 3D microstructures. More specifically, microstructures have been shown to improve cell proliferation and differentiation when they provide specific geometries, surface features and porosity. Regarding the geometry, results indicate that not only the length scale, but also the shape of the microstructures greatly affects the cell attachment and migration. Microstructured surfaces can be engineered to specifically promote cell adhesion or repellency. As for porosity, the results show that there are specific porosity intervals that provide optimal cell proliferation and differentiation. As such, optimized geometries have been shown to induce stronger osteogenic differentiation (1.5 times higher ALP activity), mineralization (1.3 times higher amount of calcified minerals) and osteocalcin secretion (2.3 times higher) [96].

Furthermore, cell behavior can be controlled effectively using dual-scale/hierarchical structures. More specifically, these are structures that present geometric features at different scales, such as micro- and nanoscales. A prevalent bio-application employing such dual-scale structures emerged as particularly interesting for their cell-repellency characteristics. This was achieved through LDW via TPP fabrication of microstructures with controlled nano-patterned surfaces. These structures have been reported to decrease cellular adhesion by ~60% as compared to flat surfaces [85].

Mechanical stimulation can also be used to enhance the effect of microstructures on the cell behavior. Recent research indicates that mechanical stimulation in tissue engineering often targets the deformation of the cellular nucleus, which is known as a highly sensitive organelle that greatly influences cell behavior. Existing results show that ultrasound stimulation of topological surfaces induces strong nuclei deformation [25,26,27,114,115,116,117,118,119], which in turn improves the osteogenic processes. For example, a 200% increase in the production of osteogenic markers, as determined from measurements of the alkaline phosphatase activity and osteocalcin production, has been reported for low-intensity pulsed ultrasound stimulation of 3D structures comprising of microarrays of vertical microtubes [27].

Materials that can be used for the fabrication of 3D microstructures for tissue engineering need to be, first and foremost, biocompatible. However, controlling the chemical composition at the surface of the structures (contact point between cells and structures) is also important, as they provide additional controllable properties that can promote specific cellular processes. A recent study presents the capabilities of the Initiated Chemical Vapor Deposition method, which has been used to coat structures with polylactic acid and acrylonitrile butadiene styrene combined with hydrophilic polymers [28]. The results, however, showed that some important disadvantages, relating directly to tissue engineering applications, emerged. For expel, the method itself proved to be complex and required a complex, multistep protocol, which lowered the fabrication efficiency and reproducibility. Moreover, the needed temperatures reached 80 degrees Celsius, which can affect the structural integrity of the microstructure. Temperature gradients can further affect the coating process due to high-temperature gradients throughout the structure, which determines an uneven coating. Using natural materials, such as collagen and chitosan, provides several important advantages such as being non-toxic to cells and promoting cellular attachment and proliferation. A simple immersion-based procedure involving IP-L780 photopolymer and collagen–chitosan blends has been reported [29]. While the results were promising and an improvement in cell proliferation has been determined, the coating method did not guarantee a uniform coating throughout the volume of the structure. A 6.5-times increase in the Osteocalcin secretion has been reported for 3D microstructures coated with a collagen–chitosan 20/80 blending ratio, as compared to the case of un-coated 3D microstructures [29].

One of the most pressing issues for implantable 3D structures is represented by post-implantation infections. They are caused by inflammatory cells that invade the contact area between the tissue and implanted structures. These infections are detrimental to tissue regeneration as they can produce fibrosis, inflammation and destruction of surrounding tissue. As such, efforts are dedicated to developing various therapeutic solutions. Recent research reports the development of 3D structures that can not only improve and control cellular processes, but they reduce local inflammation through controlled drug delivery. Structures’ activation for releasing drugs in a spatially and temporally controlled manner drug delivery can be achieved using various stimuli (light, ultrasounds, magnetic fields, etc.). Among these, electric stimulation seems to be favored due to the fact that it can improve the cell adhesion, proliferation and differentiation of various cell types, such as cells for the nervous system, skin, bone and muscles. Particular studies on the controlled release of dexamethasone have been conducted, since it is a highly employed and widely used anti-inflammatory drug [137,138,139]. Recent results indicate that LDW via the TPP technique was successful in fabricating arrays of vertical microtubes that were used as microreservoirs for the drug; the electrical stimulation was achieved by mixing the drug with a biocompatible and electrically conductive polymer, polypyrrole; the electrically stimulated samples enabled a 98% release of dexamethasone at the end of the stimulation protocol; and the osteogenic efficiency of these systems showed a 220% increase in alkaline phosphatase activity [30].

Among other types of stimuli used, with more or less success, to control cell behavior or to improve cellular processes, magnetic stimulation is often employed for accelerating these processes, as it was shown that, in vivo, the regeneration processes require a significant amount of time. In particular, static magnetic fields were studied to accelerate the proliferation, migration, orientation and differentiation of osteoblast-like cells, as well as to stimulate the osteogenic differentiation in bone marrow-derived mesenchymal stem cells [31,140,141]. In order to achieve that, recent research generally reports the incorporation of magnetic nanoparticles into biomaterials, with a strong focus on superparamagnetic nanoparticles, due to a series of advantages they offer in the context of tissue engineering (improved cell adhesion, differentiation and in vivo tissue formation, no cytotoxicity, promotes tissue growth, magnetic activation only in the presence of an external magnetic field, etc.). Despite the promising results, the studies regarding magnetically active 3D structures are limited due to the difficulty of fabricating reproducible microstructures with incorporated magnetic nanoparticles. Recent research shows that implantable magnetically active 3D microstructures are most often obtained through immersion-based fabrication processes, with more recent research presenting other fabrication methods such as spin coating or incorporating nanoparticles into liquid photoresists before laser processing. Structures fabricated using LDW via TPP using a composite made by simple physical mixing of superparamagnetic nanoparticles with a biocompatible photopolymer have been reported to show a 240% increase in the Alizarin Red Staining fluorescence intensity, which suggests an increase in the mineral deposits. Static magnetic field stimulation of the cell-seeded structures determined an increase in mineral deposits of up to 50% [140].

Overall, the recent advances in the fabrication and functionalization of 3D micro/nanostructures show promising results for tissue engineering applications, with an emphasis on solving the most pressing reproducibility and resolution issues. New technologies, such as LDW via TPP, were able to overcome some major obstacles in 3D microstructure fabrication, as well as to open new research perspectives. Improvements in the cellular processes for tissue regeneration have been obtained for all stimuli types discussed in this review (topographic, mechanical, chemical, electric and magnetic). The unique capabilities of LDW via TPP technique to produce reproducible, complex 3D structures, with sub-micrometric accuracy, combined with the actuation of these structures using the above-mentioned types of stimuli, certainly requires additional efforts to be made, in order to obtain comprehensive and reproducible results that can further pave the way for their use in vivo.

To conclude, this paper reviews the state-of-the-art stimuli-actuated 3D micro/nanostructures for tissue engineering and highlights the potential of laser-direct writing techniques for the fabrication of the structures. The paper opens up new perspectives for the use of stimuli-actuated 3D structures in a broad range of applications, such as medicine, chemistry and micro- and nanofluidics.
